# Hepatic MDM2 Causes Metabolic Associated Fatty Liver Disease by Blocking Triglyceride‐VLDL Secretion via ApoB Degradation

**DOI:** 10.1002/advs.202200742

**Published:** 2022-05-07

**Authors:** Huige Lin, Lin Wang, Zhuohao Liu, Kekao Long, Mengjie Kong, Dewei Ye, Xi Chen, Kai Wang, Kelvin KL Wu, Mengqi Fan, Erfei Song, Cunchuan Wang, Ruby LC Hoo, Xiaoyan Hui, Philip Hallenborg, Hailong Piao, Aimin Xu, Kenneth KY Cheng

**Affiliations:** ^1^ Department of Health Technology and Informatics The Hong Kong Polytechnic University Hung Hom Kowloon Hong Kong; ^2^ The State Key Laboratory of Pharmaceutical Biotechnology The University of Hong Kong Pokfulam Hong Kong; ^3^ Department of Medicine The University of Hong Kong Pokfulam Hong Kong; ^4^ Department of Neurosurgery Shenzhen Hospital Southern Medical University Shenzhen 518000 P. R. China; ^5^ Key Laboratory of Glucolipid Metabolic Diseases of the Ministry of Education Guangdong Pharmaceutical University Guangzhou 510000 P. R. China; ^6^ Department of Metabolic and Bariatric Surgery The First Affiliated Hospital of Jinan University Guangzhou 510000 P. R. China; ^7^ Department of Pharmacology and Pharmacy The University of Hong Kong Pokfulam Hong Kong; ^8^ Department of Biochemistry and Molecular Biology University of Southern Denmark Southern Denmark 5230 Denmark; ^9^ Dalian Institute of Chemical Physics Chinese Academy of Sciences Dalian 116000 P. R. China

**Keywords:** apolipoprotein B (ApoB), murine double minute 2 (MDM2), metabolic associated fatty liver, obesity, triglyceride‐VLDL

## Abstract

Dysfunctional triglyceride‐very low‐density lipoprotein (TG‐VLDL) metabolism is linked to metabolic‐associated fatty liver disease (MAFLD); however, the underlying cause remains unclear. The study shows that hepatic E3 ubiquitin ligase murine double minute 2 (MDM2) controls MAFLD by blocking TG‐VLDL secretion. A remarkable upregulation of MDM2 is observed in the livers of human and mouse models with different levels of severity of MAFLD. Hepatocyte‐specific deletion of MDM2 protects against high‐fat high‐cholesterol diet‐induced hepatic steatosis and inflammation, accompanied by a significant elevation in TG‐VLDL secretion. As an E3 ubiquitin ligase, MDM2 targets apolipoprotein B (ApoB) for proteasomal degradation through direct protein–protein interaction, which leads to reduced TG‐VLDL secretion in hepatocytes. Pharmacological blockage of the MDM2‐ApoB interaction alleviates dietary‐induced hepatic steatohepatitis and fibrosis by inducing hepatic ApoB expression and subsequent TG‐VLDL secretion. The effect of MDM2 on VLDL metabolism is p53‐independent. Collectively, these findings suggest that MDM2 acts as a negative regulator of hepatic ApoB levels and TG‐VLDL secretion in MAFLD. Inhibition of the MDM2‐ApoB interaction may represent a potential therapeutic approach for MAFLD treatment.

## Graphical abstract

Dysregulated triglyceride‐very low‐density lipoprotein (TG‐VLDL) secretion contributes to metabolic‐associated fatty liver disease. This study identifies murine double minute 2 (MDM2) as a novel E3 ubiquitin ligase that targets apolipoprotein B (ApoB) for proteasomal degradation, leading to impaired TG‐VLDL secretion and hepatic TG accumulation in obesity. Pharmacological inhibition of MDM2‐ApoB interaction by Nutlin‐3a alleviates dietary‐induced hepatic steatohepatitis.

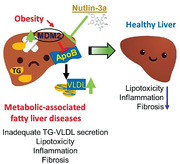

## 1 Introduction

Obesity is a major risk factor for metabolic‐associated fatty liver disease (MAFLD) and positively correlates with the severity of MAFLD, ranging from simple steatosis to steatohepatitis and its related cirrhosis and hepatocellular carcinoma.^[^ ^1^, ^2^ ^]^ MAFLD is an updated and more accurate nomenclature that was recently proposed by The International Consensus Panel to replace nonalcoholic fatty liver disease (NAFLD) and nonalcoholic steatohepatitis (NASH).^[^ ^3^ ^]^ The progression of MAFLD is a multiple‐hit process, where steatosis is the “first hit” that worsens with disease progression. Hepatic triglyceride levels are maintained by the balance between lipid input (uptake of nonesterified free fatty acids from circulation and de novo lipogenesis) and output (fatty acid oxidation and secretion of triglyceride‐rich very‐low‐density lipoprotein [TG‐VLDL]). Lipid homeostasis is disrupted in obesity, which leads to hepatic steatosis. Obese individuals with simple steatosis usually have compensatory upregulation of hepatic mitochondrial fatty acid oxidation.^[^ ^4^ ^]^ However, obese individuals with severe steatohepatitis display a significant reduction in mitochondrial oxidation and VLDL synthesis and secretion.^[^ ^5^, ^6^ ^]^

Apolipoprotein B (ApoB) and microsomal triglyceride transfer protein (MTTP) are key regulators of TG‐VLDL biosynthesis and secretion. Reduced ApoB expression causes metabolic‐associated steatohepatitis.^[^ ^6^ ^]^ Impairment of VLDL synthesis and secretion, induced by genetic mutations in *Mttp* and/or *APOB* in hypobetalipoproteinemia or by pharmacological compounds that inactivate MTTP/ApoB are associated with simple steatosis, steatohepatitis, cellular stress, and elevated alanine transaminase (ALT) levels in humans and rodents.^[^ ^6^, ^7^, ^8^, ^9^, ^10^ ^]^ In contrast, the promotion of VLDL synthesis and export by hepatic overexpression of MTTP alleviate steatohepatitis in inbred fatty liver Shionogi mice.^[^ ^11^ ^]^ Similarly, mitochondrial fatty acid oxidation and TG‐VLDL secretion induced by hepatic overexpression of peroxisome proliferator‐activated receptor gamma coactivator‐1 beta (PGC‐1*β*) protect mice against diet‐induced steatohepatitis.^[^ ^12^ ^]^ Restoration of phosphatidylcholine and subsequent TG‐VLDL assembly by inhibiting glutaminase 1 also alleviates MAFLD in a rodent model fed with a methionine‐deficient diet.^[^ ^13^ ^]^ However, the underlying cause of dysregulated TG‐VLDL and ApoB metabolism in obesity‐related MAFLD remains poorly understood.

The E3 ubiquitin ligase murine double minute 2 (MDM2) is commonly amplified in different types of cancer, including hepatocellular carcinoma, where it leads to loss‐of‐function of the tumor suppressor p53.^[^ ^14^ ^]^ Apart from its conventional role in cancer biology, the MDM2‐p53 signaling axis is essential for lipid and glucose homeostasis.^[^ ^15^, ^16^, ^17^, ^18^ ^]^ MDM2 mediates the suppressive effect of insulin on gluconeogenesis by promoting protein degradation of forkhead box protein O1 (FoxO1) in hepatocytes.^[^ ^17^ ^]^ *MDM2* mutation at residue 305 from cysteine to phenylalanine (*MDM2* ^C305F^), which disrupts its binding ability to ribosomal proteins, abolishes p53‐induced fatty acid oxidation in the liver under starvation.^[^ ^16^ ^]^ In contrast, *MDM2* ^C305F^ mutation leads to increased energy expenditure through p53 inhibition in adipose tissues.^[^ ^16^ ^]^ Loss of MDM2 in adipocytes triggers p53‐induced apoptosis and senescence, resulting in gradual loss of adipose tissue,^[^ ^18^ ^]^ whereas its deletion in pancreatic *β*‐cells suppresses pyruvate carboxylase expression in a p53‐dependent manner, leading to reduced production of coupling factors in mitochondria for insulin secretion.^[^ ^15^ ^]^ In this study, we reveal a novel detrimental role of hepatic MDM2 in the progression of MAFLD by disrupting TG‐VLDL metabolism. Under obese conditions, hepatic MDM2 overexpression leads to proteasomal degradation of ApoB, thereby lowering TG‐VLDL output, resulting in hepatic steatosis, oxidative stress, and inflammation. Importantly, pharmacological inhibition of MDM2 alleviates dietary‐induced MAFLD in an ApoB‐dependent but p53‐independent manner.

## 2 Results

### 2.1 Hepatocyte‐Specific Deletion of MDM2 Protects Against Dietary‐Induced MAFLD

**Figure 1:**
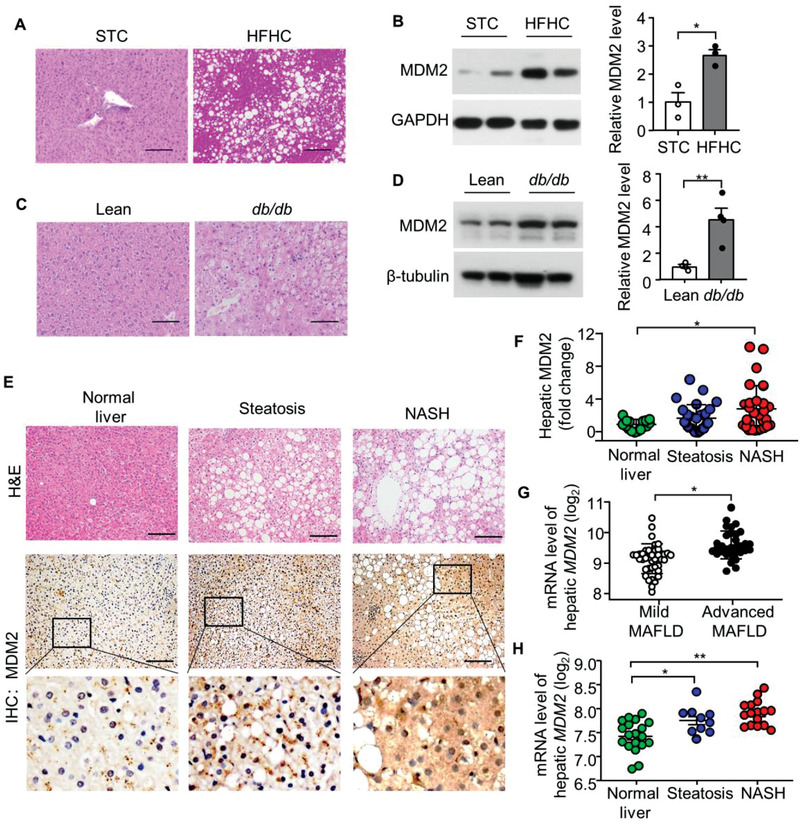
Hepatic expression of MDM2 is augmented in mouse models and humans with MAFLD. A,B) Male C57BL/6J mice fed a STC or HFHC diet for 16 weeks were used. A) Hematoxylin and Eosin (H&E) staining of liver sections. B) Immunoblotting analysis of MDM2 and GAPDH in liver lysates. The right panel is densitometric analysis for the relative abundance of MDM2 normalized with GAPDH (*n* = 3). C,D) 12‐week‐old male *db*/*db* obese mice and their lean controls were used. C) H&E staining of liver sections. D) Immunoblotting analysis of MDM2 and *β*‐tubulin in liver lysates. The right panel is densitometric analysis of MDM2 normalized with *β*‐tubulin (*n* = 4). E) Liver sections of morbidly obese humans with simple steatosis (Steatosis; *n* = 29) or steatosis, inflammation, and fibrosis (NASH; *n* = 28) or no aforementioned pathological change (Normal liver; *n* = 16) were subjected to H&E staining and immunohistochemical (IHC) staining of MDM2. F) Quantification of the relative expression of MDM2 in the three groups. G) Hepatic mRNA levels of *MDM2* in human with different degrees of MAFLD. Microarray data GSE49541 was downloaded from the Gene expression omnibus database. Mild MAFLD (fibrosis stage 0–1; *n* = 40); Advanced MAFLD (fibrosis stage 3–4; *n* = 32). H) Hepatic mRNA levels of *MDM2* in human with clinically defined normal (*n* = 19), steatosis (*n* = 10), and NASH (*n* = 16). Dataset was download from ArrayExpress public repository (Accession number: E‐MEXP‐3291) Scale bar: 100 *μ*m. Representative images are shown. All data are presented as mean ± SEM. *p < 0.05 and **p < 0.01. (Two‐tailed independent Student's *t*‐test in panel B,D,G) and Kruskal Wallis test in panel F), and One‐way ANOVA with Tukey test for panel H).

To investigate the pathophysiological relevance of MDM2 in MAFLD, we first examined MDM2 expression in liver samples isolated from high‐fat and high‐cholesterol (HFHC) diet‐induced and leptin receptor‐deficient (*db/db)* obese mice. Both the obese mouse models exhibited hepatic steatosis, accompanied by elevated hepatic MDM2 protein levels when compared with their corresponding standard chow (STC)‐fed controls and lean (*db/+*) controls (**Figure** **1**A–D). To explore the clinical relevance of MDM2, we measured hepatic MDM2 levels in obese human subjects without or with different degrees of MAFLD. According to the histological scoring system developed by NASH Clinical Research Network, the subjects were divided into three groups as follows: 1) obesity without MAFLD (Normal liver), 2) obesity with simple steatosis (Steatosis), and 3) obesity with hepatic inflammation and/or fibrosis (NASH) (Figure 1E; and Table S1, Supporting Information). Immunohistochemical analysis revealed that hepatic MDM2 was significantly upregulated in obese humans with NASH compared with those without MAFLD (Figure 1E,F; and Table S1, Supporting Information). In addition, we examined hepatic *MDM2* expression in humans with different degrees of MAFLD, using the transcriptome dataset GSE49541^[^ ^19^ ^]^ and E‐MEXP‐3291.^[^ ^20^ ^]^ Analysis of GSE49541 dataset showed that hepatic mRNA expression of *MDM2* was higher in MAFLD subjects with advanced fibrosis than in those with mild fibrosis (Figure 1G). Analysis of E‐MEXP‐3291 dataset showed that mRNA expression of hepatic *MDM2* was increased in humans with steatosis and NASH compared with that in normal individuals (Figure 1H). These data suggested that hepatic MDM2 is upregulated in humans and rodent models with MAFLD.

**Figure 2:**
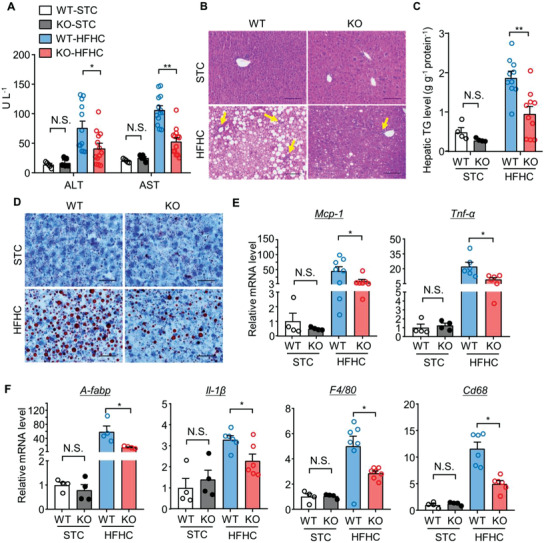
Hepatocyte‐specific deletion of MDM2 prevents diet‐induced hepatic steatosis and inflammation. 24‐week‐old male H‐MDM2‐KO mice and their WT littermates fed STC or HFHC diet were used. A) Serum levels of ALT and AST (*n* = 5 for the STC and *n* = 12–13 for the HFHC group). B) H&E staining of liver sections. Yellow arrows indicate immune cell clusters. (Scale bar: 100 µm). C) Hepatic triglyceride (TG) content normalized with protein concentration (*n* = 4 for the STC and *n* = 10 for the HFHC group). D) Oil‐red‐O staining of liver sections (scale bar: 100 *μ*m). E,F) mRNA levels of E) *Mcp‐1, Tnf‐α*, F) *A‐fabp, Il‐1β, F4/80*, and *Cd68* normalized with 18s in livers (*n* = 4 for STC and *n* = 4–8 for HFHC groups). The mRNA levels are expressed as fold change over WT‐STC. Representative histological images are shown. All data are presented as mean ± SEM. *p < 0.05 and **p < 0.01. (U test for STC in panel C), and *Tnf‐α* in panel E), and *A‐fabp, F4/80* in panel F); Welch's *t*‐test for *Mcp‐1* in panel E) and *Cd68* in panel F); two‐tailed independent Student's *t*‐test for the remaining data). Not statistical significance (N.S.).

To determine the impact of hepatic deletion of MDM2 on MAFLD development, we generated a mouse model lacking MDM2 in hepatocytes (H‐MDM2‐KO mice). Approximately, 60% reduction in MDM2 and 400% induction in p53 protein expression were observed in the livers of H‐MDM2‐KO mice (Figure S1A, Supporting Information). The residual MDM2 protein may have been derived from nonhepatocytes in the livers of H‐MDM2‐KO mice. Eight‐week‐old male H‐MDM2‐KO mice and their WT littermates were fed a STC or HFHC diet to induce obesity and its associated hepatic manifestations. There were no differences in serum markers of liver injury, aspartate aminotransferase (AST), and ALT between H‐MDM2‐KO mice and WT controls fed STC, whereas HFHC diet‐induced liver injury was significantly alleviated by hepatic deletion of MDM2 (**Figure** **2**A). After 16 weeks of dietary treatment, under STC feeding hepatic lipid content of H‐MDM2‐KO mice was unchanged from that of WT controls, but liver‐specific deletion of MDM2 prevented HFHC diet‐induced hepatic steatosis (Figure 2B–D). Real‐time quantitative PCR (qPCR) analysis revealed that the HFHC diet‐induced expression of proinflammatory cytokines, such as tumor necrosis factor‐alpha (*Tnf‐α*), interleukin 1 beta (*Il‐1β*), monocyte chemoattractant protein‐1 (*Mcp‐1*), adipocyte‐fatty acid binding protein (*A‐fabp*), and macrophage‐specific markers *F4/80* and *Cd68*, were less pronounced in H‐MDM2‐KO mice than in WT controls (Figure 2E,F). In line with the changes in hepatic inflammatory markers, the oxidative stress marker malondialdehyde (MDA) decreased in H‐MDM2‐KO mice compared to WT controls under HFHC diet feeding, while the circulating levels of MCP‐1 and adiponectin were similar between the two genotypes (Figure S1B–D, Supporting Information). Hepatic oxidative stress was lower in HFHC diet‐fed H‐MDM2‐KO mice than in their WT controls (Figure S1E,F, Supporting Information). In contrast, hepatic deletion of MDM2 had no obvious effect on cellular apoptosis or proliferation in the liver (Figure S2A–D, Supporting Information). In addition, energy expenditure, body weight, lean mass, and fat mass were similar between KO and WT mice (Figure S3, Supporting Information). Collectively, these data indicate that H‐MDM2‐KO mice are resistant to HFHC diet‐induced upregulation of hepatic steatosis and injury.

### 2.2 MDM2 Deficiency Increases TG‐VLDL Secretion in Mice

**Figure 3:**
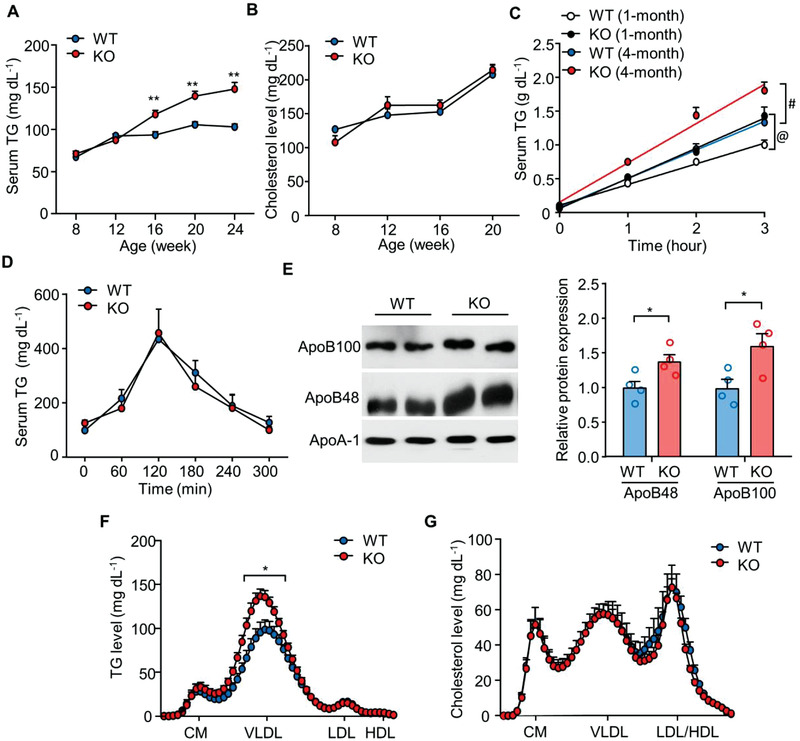
Abrogation of MDM2 in hepatocytes promotes TG‐VLDL secretion. H‐MDM2‐KO and WT littermates fed HFHC diet were used. Serum levels of A) triglyceride (TG) and B) cholesterol under ad libitum feeding (*n* = 7–10). C) Serum TG levels after tyloxapol injection in the 16 h fasted mice fed HFHC diet for 1 and 4 months (*n* = 4–5). @ *p* < 0.05, WT (1 month) vs KO (1 month); # *p* < 0.05; WT (4 month) vs KO (4 month) (fold change of TG‐VLDL secretion rate). D) Serum TG levels after oral gavage with olive oil in the mice fed HFHC diet for 3 months (*n* = 5). E–G) Serum samples collected from the mice fed HFHC diet for 4 months after injection with tyloxapol for 3 h were analyzed. E) Immunoblotting analysis of ApoB100, ApoB48, and ApoA‐1 in the serum. The bar chart is the densitometric quantification of ApoB48 and ApoB100 normalized with ApoA‐1. Representative immunoblot images are shown (*n* = 4). Distribution of F) TG and G) cholesterol in different densities of lipoproteins in the serum samples (CM: chylomicron; VLDL: very low density lipoprotein; LDL: low density lipoprotein; HDL: high density lipoprotein). All data are presented as mean ± SEM. *p < 0.05 and **p < 0.01 (Two‐tailed independent Student's *t*‐test).

Given that dysregulation of hepatic lipid metabolism contributes to MAFLD, we examined the lipid profiles of H‐MDM2‐KO mice. Since we observed no differences in several hepatic parameters, including inflammation, lipid metabolism, and injury markers between H‐MDM2‐KO mice and WT controls fed STC, we only examined the animals fed the HFHC diet in subsequent experiments unless stated otherwise. Hepatic deletion of MDM2 gradually and modestly increased circulating levels of TG after HFHC diet feeding for 8 weeks and thereafter, and this increase was only observed in the fed state but not under fasting condition (**Figure** **3**A; and Table S2, Supporting Information). There were no differences in the levels of circulating cholesterol and free fatty acid (FFA) under fasting or fed conditions between the two genotypes (Figure 3B; and Table S2, Supporting Information), whereas fasting‐induced ketogenesis (as exemplified by circulating *β*‐hydroxybutyrate) was lower in H‐MDM2‐KO mice than in WT controls (Table S2, Supporting Information). We further evaluated the role of hepatic MDM2 in the regulation of TG‐VLDL metabolism. To monitor hepatic TG‐VLDL secretion, HFHC diet‐fed H‐MDM2‐KO mice and their WT controls were intravenously injected with tyloxapol, a lipase inhibitor that inhibits TG‐VLDL catabolism in peripheral tissues. Upon tyloxapol administration, circulating TG levels increased dramatically in WT controls, which was further augmented by genetic deletion of MDM2 (Figure 3C). In contrast, H‐MDM2‐KO mice and WT controls displayed similar clearance of circulating TG after oral gavage with olive oil (Figure 3D). These findings suggest that hepatic MDM2 regulates TG‐VLDL secretion, but not TG clearance. Consistent with these findings, circulating levels of ApoB, a major structural component of VLDL particles, were elevated in H‐MDM2‐KO mice after tyloxapol injection (Figure 3E). In obese human subjects with NASH, hepatic MDM2 levels significantly correlated with circulating ApoB levels (*r* = −0.5068, *p* = 0.0059) (Figure S4 and Table S1, Supporting Information), suggesting that MDM2 is inversely associated with TG‐VLDL in MAFLD. Fractionation of circulating lipoproteins into chylomicron (CM), VLDL, low‐density lipoprotein (LDL), and high‐density lipoprotein (HDL) using high‐performance liquid chromatography revealed that only TG in the VLDL fraction was significantly higher in H‐MDM2‐KO mice after injection with tyloxapol (Figure 3F). Surprisingly, no change in cholesterol levels was observed in different lipoprotein fractions (Figure 3G). Taken together, hepatic deletion of MDM2 alleviates MAFLD, probably through the induction of TG‐VLDL secretion.

### 2.3 MDM2 Acts as an E3 Ubiquitin Ligase Targeting ApoB for Proteasomal Degradation

**Figure 4:**
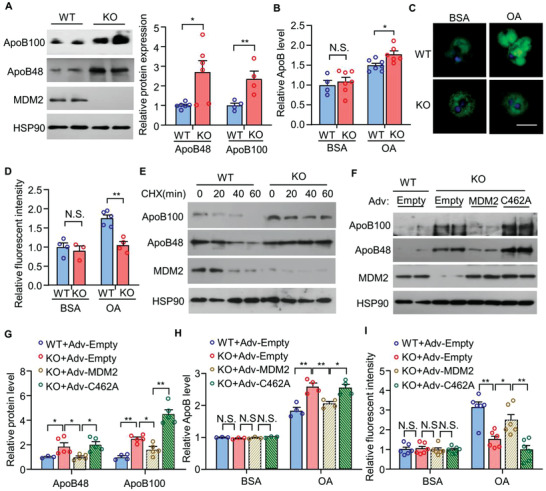
The E3 ligase activity of MDM2 is indispensable for its suppressive effects on ApoB expression and secretion in primary hepatocytes under obese condition. Primary hepatocytes were isolated from H‐MDM2‐KO and WT littermates fed the HFHC diet for 12 weeks. A–D) Primary hepatocytes were treated with 0.4 × 10^−3^ m oleic acid (OA) conjugated with BSA or BSA as control for 8 h, and subjected to A) immunoblotting analysis of ApoB100, ApoB48, MDM2, and HSP90. The bar chart is relative expression levels of ApoB100 and ApoB48 normalized with HSP90 (*n* = 4–6). B) The relative ApoB levels in the culture medium (*n* = 4–7). C) The cells were fixed and stained with BODIPY lipid probes (green) and DAPI (blue) (scale bar: 10 *μ*m). D) The relative intensity of BODIPY green fluorescence over cell size (*n* = 3–5) in panel C). Each data point represents the average BODIPY intensity in an area of interest. E) Primary hepatocytes were treated with cycloheximide (CHX; 50 *μ*g mL^−1^) for different time points, followed by immunoblotting analysis as indicated. F–I) Primary hepatocytes were infected with adenovirus encoding empty control, wild‐type (WT) MDM2, or its E3 ligase defective mutants C462A for 48 h. The infected hepatocytes were treated with 0.4 × 10^−3^ m OA or BSA for 8 h, and then subjected to F) immunoblotting analysis. G) The bar chart is densitometric analysis of hepatic ApoB normalized with HSP90 in panel F (*n* = 3–5). H) The relative ApoB levels in culture medium normalized with protein concentration (*n* = 3–4). I) BODIPY lipid staining (*n* = 6). Representative images are shown. All data are presented as mean ± SEM. *p < 0.05 and **p < 0.01. (Two‐tailed independent Student's *t*‐test in panel A,B,D) or One Way ANOVA with Tukey test in panel G–I). Not statistical significance (N.S.).

We further investigated the underlying mechanism behind elevated TG‐VLDL secretion led by MDM2 deficiency. We measured the expression of genes involved in lipogenesis [sterol regulatory element‐binding protein 1 (*Srecbp‐1c*), fatty acid synthase (*Fasn*), stearoyl‐CoA desaturase‐1 (*Scd1*), and diacylglycerol acyltransferase‐2 (*Dgat2*)]; fatty acid oxidation [*lipin‐1*, *Cd36*, carnitine palmitoyltransferase I (*Cpt1α*), peroxisome proliferator‐activated receptor‐alpha (*Pparα*), *Pgc‐1α*, medium‐chain acyl‐CoA dehydrogenase (*Mcad*), and fibroblast factor 21 (*Fgf21* )]; ketogenesis [acetyl‐CoA acetyltransferase 1 (*Acat1*), 3‐hydroxy‐3‐methylglutaryl‐CoA synthase 2 (*Hmgcs2*), 3‐hydroxy‐3‐methylglutaryl‐CoA lyase (*Hmgcl*), and 3‐hydroxybutyrate dehydrogenase 1 (*Bdh*1)]; and VLDL metabolism (*ApoB* and *Mttp*). However, no changes were observed in H‐MDM2‐KO mice compared to their WT controls (Figure S5A–D, Supporting Information). In agreement with elevated circulating ApoB levels, the hepatic expressions of ApoB48 and ApoB100 proteins were mildly but significantly induced in HFHC diet‐fed H‐MDM2‐KO mice (Figure S5E, Supporting Information). In contrast, similar levels of Mttp, Ppar*α*, Pgc1*α*, Hmgcs2, and Hmgcl were found in the livers of both genotypes (Figure S5E, Supporting Information). Intrigued by the result showing that hepatic MDM2 deficiency leads to an induction of ApoB protein without affecting its mRNA level (Figure S5D, Supporting Information), we explored whether MDM2 regulates ApoB levels in post‐translational pathways. Notably, the majority of unlipidated ApoB undergoes degradation, and pathways regulating this degradation are regarded as the key determinants of intracellular ApoB levels and thereby TG‐VLDL secretion rate.^[^ ^21^, ^22^ ^]^ To further confirm the in vivo findings, we examined the effect of MDM2 downregulation on ApoB metabolism using primary hepatocytes isolated from H‐MDM2‐KO mice fed HFHC diet and HepG2 cells. HepG2 cells resemble human hepatocytes in ApoB biosynthesis and TG‐VLDL secretion.^[^ ^23^ ^]^ Consistent with our observations in the liver, MDM2 *null* primary hepatocytes and HepG2 cells transfected with siRNA against *MDM2* displayed an increase in ApoB protein expression when compared with that in their respective control cells (**Figure** **4**A; and Figure S6A,B, Supporting Information). To investigate whether MDM2 controls the stability of ApoB, we treated hepatocytes with cycloheximide, a protein synthesis inhibitor, and observed a significant increase in ApoB stability in MDM2 *null* hepatocytes and HepG2 cells transfected with siRNA against *MDM2* when compared with that in their respective control cells (Figure 4E; and Figure S6C, Supporting Information). Downregulation of MDM2 expression resulted in a reduction of intracellular lipid levels, accompanied by increased ApoB secretion in HepG2 cells and primary hepatocytes upon stimulation with oleic acid (OA, an unsaturated acid known to promote TG‐VLDL and ApoB secretion^[^ ^24^, ^25^ ^]^) (Figure 4B–D; and Figure S6D–F, Supporting Information). Consistently, [^3^H] glycerol labeling assay revealed that *MDM2* knockdown increased TG secretion in HepG2 cells in the presence of oleic acid (Figure S6G, Supporting Information). Notably, siRNA‐mediated MDM2 downregulation did not alter proliferation or apoptosis in HepG2 cells (Figure S7A,B, Supporting Information).

**Figure 5:**
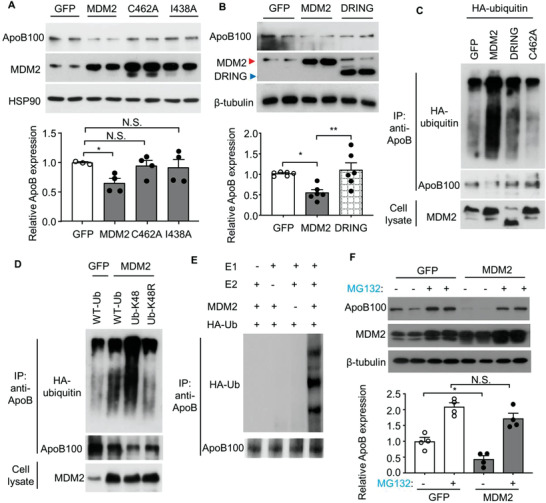
The E3 ubiquitin ligase MDM2 induces proteasomal degradation and K48‐linked ubiquitination of ApoB in HepG2 cells. A,B) HepG2 cells were transfected with plasmids encoding for wild‐type (WT) MDM2 or its E3 ligase defective mutants including C462A or I438A mutant, or truncated MDM2 without RING finger domain (DRING) or GFP control for 48 h, followed by immunoblotting analysis. The bar charts in the lower panels are densitometric analyses of ApoB normalized with HSP90 or *β*‐tubulin (*n* = 3–6). C) HepG2 cells were transfected with plasmids encoding HA‐ubiquitin and WT‐MDM2 or its E3 ligase defective mutants or GFP control as indicated for 48 h. D) HepG2 cells were transfected with plasmids expressing MDM2 or GFP, and WT‐Ub, Ub‐K48, or Ub‐K48R mutant as indicated for 48 h. C,D) The transfected cells were subjected to immunoprecipitation (IP) using an anti‐ApoB antibody, followed by immunoblotting as indicated. E) In vitro ubiquitination assay. The recombinant E1 and E2 enzymes, MDM2, HA‐tagged ubiquitin (Ub) proteins, and native ApoB protein were added to the ubiquitination reaction mix, followed by IP of ApoB and immunoblotting analysis as indicated. F) Immunoblotting analysis of ApoB and MDM2 in HepG2 cells transfected with WT MDM2 or GFP with or without MG132 (10 × 10^−6^ m) for 6 h. The lower panel is densitometric analysis of ApoB100 normalized with *β*‐tubulin (*n* = 4). All data are presented as mean ± SEM. *p < 0.05 and **p < 0.01. (One‐way ANOVA with Tukey test for panel B) and F) or two‐tailed independent Student's *t*‐test for panel A). N.S. (Not significant).

In contrast, MDM2 overexpression drastically reduced ApoB protein expression in HepG2 cells (**Figure** **5**A) without altering cell proliferation or apoptosis (Figure S7C,D, Supporting Information). To determine whether the E3 ligase activity of MDM2 is responsible for its suppressive action on ApoB, we examined the effects of the following MDM2 mutations on ApoB protein levels: 1) deletion of the RING domain (DRING); 2) mutation of cysteine^462^ in the RING domain to alanine (C462A), which leads to misfolding of the RING domain; and 3) replacement of isoleucine^438^ with alanine (I438A), which abolishes the binding of E2 ubiquitin‐conjugating enzyme. Ectopic expression of MDM2 mutants with defective E3 ligase activity did not reduce ApoB protein levels in HepG2 cells (Figure 5A,B). To further confirm these findings, we isolated primary hepatocytes from HFHC diet‐fed H‐MDM2‐KO mice and their WT controls, followed by an infection of adenovirus encoding wild‐type MDM2, MDM2‐C642A mutant, or empty control. Adenovirus‐mediated replenishment of wild‐type MDM2, but not MDM2‐C642A mutant, suppressed the elevated expression and secretion of ApoB, and increased lipid accumulation in MDM2 *null* hepatocytes (Figure 4F–I).

Overexpression of MDM2, but not its mutants with defective E3 ligase activity, increased lysine48‐linked ubiquitination of ApoB in HepG2 cells for proteasomal degradation (Figure 5C,D). An in vitro assay confirmed that ApoB ubiquitination increased in the presence of MDM2 (Figure 5E). Treatment with the proteasome inhibitor MG132 abolished the suppressive effect of overexpressed MDM2 on ApoB expression in HepG2 cells (Figure 5F). Collectively, these data suggest that MDM2 acts as a novel E3 ubiquitin ligase that targets ApoB for proteasomal degradation.

### 2.4 Loss of MDM2 Increases ApoB Expression and TG‐VLDL Secretion Independent of p53

**Figure 6:**
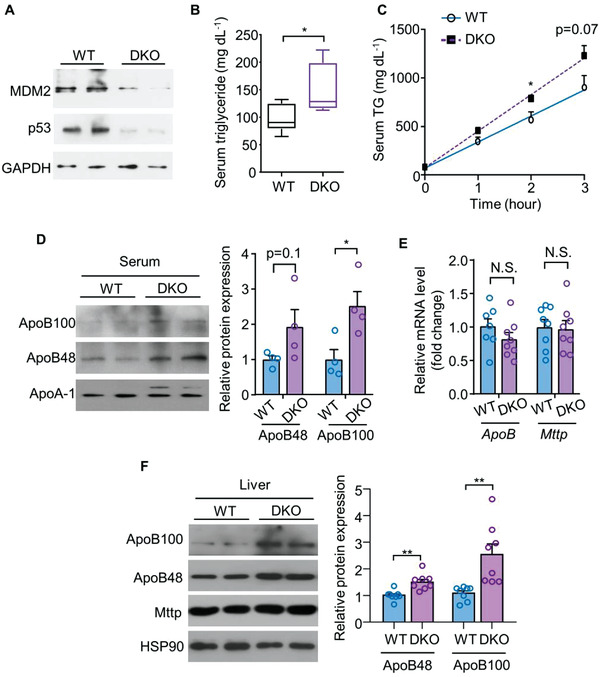
MDM2 regulates TG‐VLDL secretion and ApoB expression independent of p53. Hepatic MDM2‐p53 double knockout (DKO) mice and their WT littermates fed HFHC diet were used. A) Immunoblotting analysis of MDM2, p53, and GAPDH in livers of the mice. B) Random fed serum TG levels in the mice fed HFHC diet for 8 weeks (*n* = 7–8). C) The mice fed HFHC diet for 8 weeks were injected with tyloxapol. Circulating levels of TG upon tyloxapol injection (*n* = 5). D) Serum samples were collected from the mice after tyloxapol injection for 3 h, followed by immunoblotting analysis of circulating ApoB100 and ApoB48. The right bar chart is densitometric analysis of circulating ApoB100 and ApoB48 normalized with ApoA‐1 (*n* = 4). E) mRNA and F) protein expressions of ApoB and Mttp in the livers of mice after HFHC feeding for 16 weeks (*n* = 7–8). Representative immunoblots are shown. All data are presented as mean ± SEM. *p < 0.05, **p < 0.01 (Welch's *t*‐test for ApoB100 in panel F); two‐tailed independent Student's *t*‐test for the remaining data). N.S. (Not significant).

We and others have recently demonstrated that the metabolic actions of MDM2 in the liver, adipose tissue, and pancreatic *β* cells are mainly mediated by p53,^[^ ^15^, ^16^, ^18^ ^]^ which controls hepatic lipid metabolism and inflammation.^[^ ^26^, ^27^, ^28^, ^29^ ^]^ To ascertain whether the changes in TG‐VLDL metabolism and ApoB expression, and improvement of MAFLD in H‐MDM2‐KO mice were due to p53 activation, we generated hepatocyte‐specific *MDM2* and *p53* double knockout (hereafter referred to DKO) mice. Immunoblot analysis revealed that the protein expression of both MDM2 and p53 were reduced in DKO mice compared to that in their WT controls (**Figure** **6**A). The residual MDM2 and p53 proteins in the livers of DKO mice are likely present because of their expressions in nonparenchymal cells. Similar to the observations in H‐MDM2‐KO mice, circulating TG levels and TG‐VLDL secretion were elevated in DKO mice compared to their WT controls (Figure 6B,C). As observed previously, increased circulating and hepatic ApoB protein levels were observed in DKO mice compared to their WT littermates, whereas mRNA levels of *ApoB* and *Mttp* remained unchanged (Figure 6D–F). The hepatic expression profiles of genes related to apoptosis and cell proliferation were similar between DKO mice and their WT littermates (Figure S2E,F, Supporting Information). Consistent with the findings in DKO mouse model, the promoting effect of MDM2 silencing on ApoB expression was not affected by concomitant silencing of *p53* in HepG2 cells (Figure S6H, Supporting Information). These results demonstrate that the direct regulation of ApoB expression and TG‐VLDL secretion by MDM2 is independent of p53.

Next, we evaluated whether the hepatoprotective effect of MDM2 deficiency requires p53 activation. DKO mice displayed similar levels of hepatic inflammation, liver injury, and lipid accumulation as those in their WT littermates fed a HFHC diet (Figure S8, Supporting Information). Compared with H‐MDM2‐KO mice and WT controls (*MDM2* ^flxoed/flxoed^ *p53* ^floxed/floxed^), DKO mice exhibited significantly higher hepatic expression of key lipogenic enzymes, including Acetyl‐CoA carboxylase 1 (Acc1), Fasn, and ATP citrate lyase (Acly) (Figure S9, Supporting Information). We speculated that the upregulation of lipogenic enzyme expression counteracted the effects of enhanced TG‐VLDL secretion in DKO mice. Therefore, the net result was no improvement of MAFLD in DKO mice.

### 2.5 MDM2 Mediates Ubiquitination and Degradation of ApoB

We hypothesized that MDM2‐induced ApoB degradation would require protein–protein interaction. Coimmunoprecipitation assay indicated that MDM2 interacted with ApoB in the liver of C57BL/6J mice, primary hepatocytes, and HepG2 cells (Figure S10B–D, Supporting Information). Consistent with the findings of previous studies,^[^ ^24^, ^25^ ^]^ oleic acid upregulated ApoB protein expression, which coincided with a reduced MDM2‐ApoB interaction (Figure S10A,D–F, Supporting Information). In addition, treatment with stearic acid also reduced MDM2‐ApoB interaction (Figure S10F, Supporting Information). In contrast, treatment with oleic or stearic acid had no obvious effects on MDM2‐p53 interaction (Figure S10G, Supporting Information). Our in silico analysis revealed two putative MDM2‐binding motifs in ApoB (Figure S11A, Supporting Information), which are similar to those identified in p53, interferon regulatory factor 1 (IRF1), IRF2, and cyclophilin B.^[^ ^30^ ^]^ Notably, binding of these proteins to MDM2 is sensitive to the MDM2 antagonist, Nutlin‐3a.^[^ ^30^ ^]^ We speculated that the interaction between ApoB and MDM2 could be prevented by Nutlin‐3a. Indeed, treatment with Nutlin‐3a disrupted the interaction between MDM2 and ApoB in hepatocytes, accompanied by increased ApoB expression and secretion and a reduced lipid accumulation (Figures S11C, S12A–C, and S13A–C, Supporting Information). The induction of ApoB100 expression by Nutlin‐3a treatment did not require p53 activation, but was abrogated in MDM2 *null* hepatocytes (Figure S13B,C, Supporting Information). The promoting effect of Nutlin‐3a on ApoB expression was also observed in mouse liver (Figure S14A, Supporting Information). However, Nutlin‐3a did not alter proliferation or apoptosis in hepatocytes (Figure S7E,F, Supporting Information).

Next, we determined whether the MDM2‐ApoB interaction was mediated by the putative MDM2 binding motifs. To this end, we generated expression vectors encoding truncated ApoB fragments [amino acid 1–624 (containing *βα*1 domain; named as ApoB‐1–624) and amino acid 3419–4018 (containing *β*2 domain; named as ApoB‐3419–4018)] that contained the two putative binding motifs. The key residues in the binding motifs of ApoB‐1–624 and ApoB‐3419–4018 fragments were mutated from L347, F348, and L351 to alanine residues (named L347A/F348A/L351A mutant) and from L3684, W3685, and L3688 to alanine residues (named L3684A/W3685A/L3688A mutant), respectively (Figure S11B, Supporting Information). HEK293 cells were co‐transfected with Myc‐tagged MDM2 and truncated ApoB or truncated ApoB with the above mutations in putative MDM2 binding motifs. Coimmunoprecipitation analysis showed that ApoB bound to MDM2 through putative binding motifs, and mutation of the key residues in the motifs largely abrogated ApoB‐MDM2 interaction (Figure S11D,E, Supporting Information). Collectively, these findings indicate that MDM2 promotes proteasomal degradation of ApoB through protein–protein interaction.

### 2.6 Pharmacological Inhibition of MDM2 Alleviates Metabolic‐Associated Steatohepatitis by Upregulation of ApoB‐Mediated TG‐VLDL Secretion

**Figure 7:**
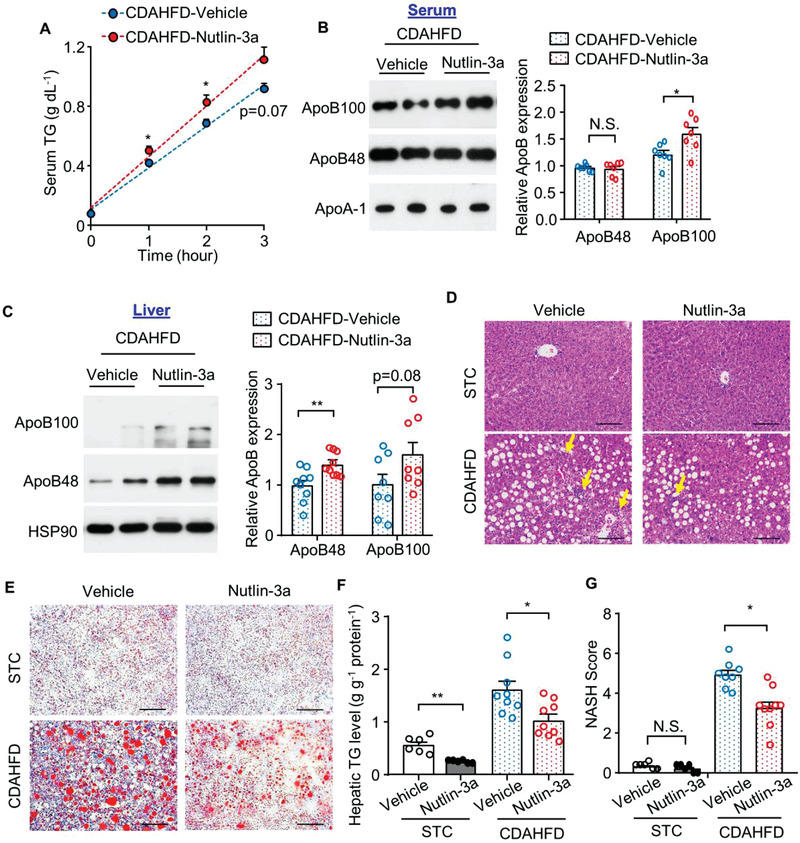
Treatment with MDM2 inhibitor increases TG‐VLDL secretion and alleviates CDAHFD‐induced steatohepatitis. C57BL/6J mice were fed CDAHFD diet for 2 weeks, followed by treatment with Nutlin‐3a or DMSO (as vehicle control) for 1 week (panel A,B) and 2 weeks (panel C–G). A,B) Serum was collected after tyloxapol injection for 3 h. Circulating levels of A) TG and B) ApoB. The bar chart in panel B) is the relative ApoB100 and ApoB48 expression normalized with ApoA‐1 (*n* = 7). C) Immunoblotting analysis of ApoB100 and ApoB48 in the liver tissues. The bar chart is densitometric quantification of ApoB proteins normalized with HSP90 (*n* = 8–9). D) Representative H&E staining and E) Oil‐red O staining of liver sections. Scale bar: 100 µm. Yellow arrows indicate immune cell clusters. F) Hepatic TG contents normalized with protein concentration (*n* = 6–9). G) NASH score (*n* = 6–9). All data are presented as mean ± SEM. *p < 0.05 and **p < 0.01. (Welch's *t*‐test for STC in panel F); two‐tailed independent Student's *t*‐test for the remaining data). Not significant (N.S.).

Currently, there are no approved therapies for metabolic‐associated steatohepatitis, the more aggressive form of MAFLD, which is characterized by severe inflammation and fibrosis. To examine the therapeutic potential of MDM2 inactivation in steatohepatitis, we injected Nutlin‐3a into C57BL/6J mice fed a choline‐deficient amino acid‐defined high‐fat diet (CDAHFD), a dietary treatment that rapidly induces steatohepatitis and progressive fibrosis in mice, at least in part, by inhibiting TG‐VLDL and ApoB secretion.^[^ ^31^, ^32^ ^]^ CDAHFD feeding was started at the age of 8 weeks, and 2 weeks later, the mice were intraperitoneally injected with Nutlin‐3a or dimethyl sulfoxide (DMSO, as vehicle control) twice per day (Figure S14B, Supporting Information). Nutlin‐3a administration had no obvious effect on body weight (Figure S14C, Supporting Information). CDAHFD drastically increased the circulating levels of AST and ALT in C57BL/6J mice injected with vehicle, but this hepatic injury was significantly alleviated by Nutlin‐3a treatment (Table S4, Supporting Information). Similar to the observations in H‐MDM2‐KO mice, treatment with Nutlin‐3a enhanced TG‐VLDL secretion, circulating TG, and hepatic levels of ApoB under CDAHFD feeding (**Figure** **7**A–C; and Table S4, Supporting Information). Nutlin‐3a treatment moderately increased blood glucose levels under STC feeding (Table S4, Supporting Information), which may be due to its negative effect on glucose‐stimulated insulin secretion, as we previously reported.^[^ ^15^ ^]^ Strikingly, hepatic steatosis (Figure 7D–F) and inflammation (Figure S14D, Supporting Information) induced by CDAHFD were significantly alleviated by Nutlin‐3a treatment. The major characteristic distinguishing advanced steatohepatitis from simple steatosis is the presence of fibrosis. To determine whether alleviation of steatosis and inflammation also improves fibrosis in mice treated with Nutlin‐3a, we measured the collagen content in liver sections by Picro Sirius Red staining. Histological visualization showed that feeding CDAHFD markedly induced collagen deposition in mice treated with vehicle, but such detrimental effect of CDAHFD was partially attenuated by Nutlin‐3a treatment (Figure S14F, Supporting Information). Expressions of fibrosis‐related genes, including connective tissue growth factor (*Ctgf*) and a family of collagen proteins, were suppressed by Nutlin‐3a treatment (Figure S14E, Supporting Information). Hepatic expression of tumor growth factor‐1 beta (*Tgf1β*) showed a decreasing tendency in CDAHFD‐fed mice treated with Nutlin‐3a (Figure S14E, Supporting Information). CDAHFD induced hepatic ballooning (a structural change of hepatic injury in steatohepatitis) and lobular inflammation, but such detrimental effects were reduced by Nutlin‐3a treatment (Figure 7D,G). In contrast, treatment with Nutlin‐3a had no effect on food intake and circulating level of *β*‐hydroxybutyrate under STC or CDAHFD condition (Table S4, Supporting Information). A modest increase in circulating cholesterol levels was observed in mice treated with Nutlin‐3a under CDAHFD‐feeding condition (Table S4, Supporting Information). Although Nutlin‐3a is known to induce apoptosis and inhibit proliferation of cancer cells,^[^ ^33^, ^34^ ^]^ this MDM2 inhibitor did not alter the apoptotic and proliferative phenomena in the liver of CDAHFD‐fed mice (Figure S15, Supporting Information).

**Figure 8:**
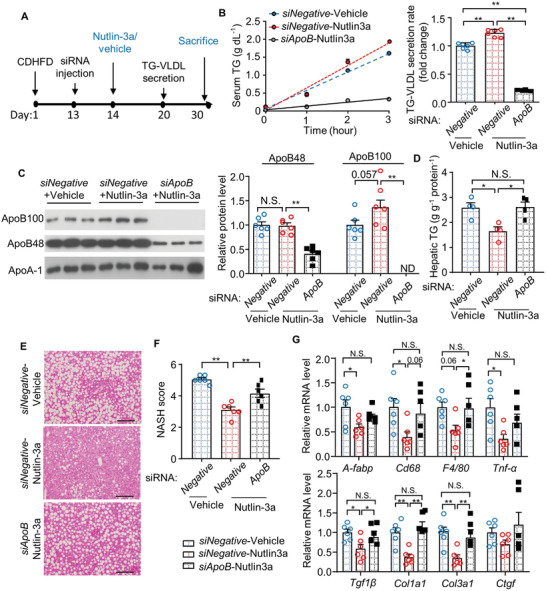
The hepato‐protective effect of Nutlin‐3a is largely attenuated by downregulation of ApoB. A) 8‐week‐old male C57BL/6J mice were fed CDAHFD for 13 days, followed by intravenous injection with siRNA against *ApoB* (*siApoB*) or Stealth siRNA negative control (*siNegative*) at day 13. The mice were then treated with Nutlin‐3a or vehicle for the remaining experimental period before sacrifice at day 30. B) In vivo TG‐VLDL secretion assay was performed at day 20. The bar chart is fold change of TG‐VLDL secretion rate over the mice treated with *siNegative* + vehicle (*n* = 6). C) Serum was collected during TG‐VLDL secretion assay, followed by immunoblotting analysis of ApoB and ApoA‐1. The right panel is densitometric analysis of ApoB100 and ApoB48 normalized with ApoA‐1 (*n* = 6). D) Hepatic TG contents determined by biochemical assay (*n* = 4). E,F) Representative images of H&E staining of liver sections. Scale bar: 100 µm. F) NASH score of liver sections in panel E) (*n* = 6). G) QPCR analysis of genes related to inflammation (upper panel) and fibrosis (lower panel) in livers (*n* = 5–6). All data are presented as mean ± SEM. *n* = 6. *p < 0.05, **p < 0.01. (Welch's ANOVA with Dunnett test for panel B); One‐way ANOVA with Tukey test for remaining panels). Not significant (N.S.).

To ascertain whether the hepatoprotective effect of Nutlin‐3a was mediated by ApoB, C57BL/6J mice fed CDAHFD were injected with siRNA against *ApoB* (*siApoB*) or Stealth siRNA negative control (*siNegative*), followed by Nutlin‐3a treatment (**Figure** **8**A). Nutlin‐3a treatment increased circulating levels of ApoB and TG‐VLDL secretion, but these changes were completely abrogated by *ApoB* knockdown (Figure 8B,C). Furthermore, the improvement of hepatosteatosis, inflammation, fibrosis, and reduction in AST and ALT levels in Nutlin‐3a treated mice were largely reversed by *ApoB* knockdown (Figure 8D–G; and Table S5, Supporting Information). These data suggest that ApoB upregulation and subsequent TG‐VLDL secretion, at least in part, are responsible for the therapeutic effect of Nutlin‐3a on MAFLD.

Since Nutlin‐3a treatment also resulted in an upregulation of p53 in the liver, we investigated whether p53 inactivation could abolish the hepatoprotective effects of Nutlin‐3a in C57BL/6J mice. To this end, we injected C57BL/6J mice with an adeno‐associated virus encoding shRNA against *p53* (AAV‐*sh‐p53*) through the tail vein (Figure S16A, Supporting Information). Previous studies have shown that complete hepatic p53 inactivation results in liver steatosis, inflammation, and/or ER stress.^[^ ^26^, ^27^, ^28^ ^]^ Therefore, we used shRNA to partially reduce hepatic p53 expression. We confirmed that injection of AAV‐*sh‐p53* reduced p53 expression only in the liver, but not in other tissues, including epididymal white adipose tissue (eWAT) and skeletal muscle (Figure S16B, Supporting Information). Immunoblot analysis showed that Nutlin‐3a treatment led to a robust increase in p53 and p21 levels, while this effect was abrogated in mice injected with AAV‐*sh‐p53* (Figure S16C, Supporting Information). Consistent with the in vitro findings, we observed that AAV‐mediated knockdown of hepatic *p53* had no impact on Nutlin‐3a‐induced upregulation of hepatic ApoB expression and its secretion into the circulation, as well as TG‐VLDL secretion in animals fed a CDAHFD (Figure S17, Supporting Information). Furthermore, AAV‐mediated *p53* silencing did not abrogate the beneficial effects of Nutlin‐3a on hepatic steatosis, inflammation, fibrosis, and liver injury (Figure S18 and Table S6, Supporting Information). Taken together, these data suggest that Nutlin‐3a improves MAFLD by upregulating ApoB expression and secretion in a p53‐independent manner.

## 3 Conclusion

MAFLD has become a major health problem worldwide owing to its high prevalence and potential progression to liver cirrhosis and cancer. Lipotoxicity mediates the development and progression of MAFLD through multiple pathways. Our study demonstrates that hepatic MDM2 is markedly amplified in obese mice with MAFLD. MDM2 inactivation alleviates hepatic steatosis, inflammation, and fibrosis in different mouse models of MAFLD. The hepatoprotective actions of MDM2 inactivation are mainly owing to its preventive effect on ApoB degradation and subsequent potentiating effect on TG‐VLDL secretion in hepatocytes, which in turn alleviates intracellular lipotoxicity. In addition, we show that MDM2 is a novel E3 ligase that targets ApoB for proteasomal degradation in the hepatocytes (Figure S19, Supporting Information).

Dysfunctional TG‐VLDL metabolism and reduced ApoB expression are linked to MAFLD. Yet the underlying cause is unknown.^[^ ^6^, ^7^, ^12^, ^13^ ^]^ Here we show that hepatic inhibition of MDM2 promotes ApoB‐mediated TG‐VLDL secretion and hence relieves steatohepatitis in rodent models. The TG‐VLDL secretion rate is modestly but significantly upregulated during the progression of obesity and MAFLD, and this regulation is more pronounced in H‐MDM2‐KO mice. This compensatory upregulation relieves hepatic lipotoxicity and oxidative stress in H‐MDM2‐KO mice. In vitro, siRNA‐mediated knockdown or genetic deletion of *MDM2* prevents OA‐induced lipid accumulation and enhances ApoB and TG secretion in hepatocytes. In addition to genetic approach, we employed the Nutlin‐3a to block MDM2‐ApoB interaction, and showed that this pharmacological approach prevents hepatic steatosis and inflammation. We believe that the improvement in steatosis is mainly caused by the direct effect of MDM2 inactivation on ApoB expression and TG‐VLDL secretion, whereas the alleviation of inflammation and fibrosis may be secondary to the relief from lipotoxicity in hepatocytes. It is also worth mentioning that inactivation of MDM2 directly inhibits inflammatory and fibrotic responses in multiple cell types; therefore, its direct action cannot be excluded.^[^ ^35^, ^36^, ^37^, ^38^ ^]^ However, genetic inactivation of MDM2 has no obvious effect on cholesterol levels, whereas Nutlin‐3a mildly increases circulating cholesterol levels. The underlying reason for these differential effects is not clear. Since the effect of MDM2 inactivation on cholesterol is modest, the potential side effects of ApoB upregulation on the cardiovascular system may be minimal, but needs to be taken into consideration.

ApoB is constitutively synthesized in hepatocytes and its expression is mainly regulated at co‐ and post‐translational levels.^[^ ^21^, ^22^ ^]^ Majority of newly synthesized ApoB undergoes nonproteasomal and proteasomal degradation. The proteasomal degradation of ApoB is tightly controlled and coordinated by several intracellular chaperons and the E3 ubiquitin ligase glycoprotein 78 (Gp78) during lipidation, intracellular translocation, and secretion of ApoB.^[^ ^22^, ^39^, ^40^ ^]^ Our in vitro and in vivo models demonstrate MDM2 as a novel E3 ligase that induces ubiquitin‐mediated proteasomal degradation of ApoB. Oleic acid reduces MDM2‐ApoB interaction. In addition, MDM2 binds to ApoB through the canonical p53‐binding pocket. Nutlin‐3a occupies the p53‐binding pocket to abrogate p53‐MDM2 interaction, and was originally developed for cancer therapy.^[^ ^33^ ^]^ Moreover, Nutlin‐3a interferes with the binding between MDM2 and hypoxia‐inducible factor‐*α* or p73 in the absence of p53.^[^ ^41^, ^42^ ^]^ In silico prediction indicates two putative binding sites of MDM2 in ApoB, and we confirm that MDM2 and ApoB interact via the binding sites L347/F348/L351 and L3684/W3685/L3688. A proteomics study revealed that the lysine residues at 2911 and 2915 of human ApoB can be ubiquitinated.^[^ ^43^ ^]^ Whether MDM2‐ApoB interaction mediated by the two binding sites contributes to ApoB ubiquitination is not known, and further investigation is warranted. In contrast, treatment with Nutlin‐3a modestly upregulates hepatic ApoB100 and ApoB48 protein expression, both in vivo and in vitro. Furthermore, our study demonstrates that proteasome inhibitor prevents MDM2‐mediated ApoB degradation. These data collectively suggest that MDM2 regulates ApoB proteasomal degradation through protein–protein interaction.

Although MDM2 regulates multiple metabolic pathways through p53,^[^ ^15^, ^16^, ^18^ ^]^ our current study reveals that MDM2 regulates lipid metabolism beyond its canonical action on p53. Upregulations of ApoB and TG‐VLDL secretion was observed in both H‐MDM2‐KO and DKO mice. Similarly, the effects of Nutlin‐3a on ApoB expression and TG‐VLDL secretion are also p53 independent. In addition to TG‐VLDL secretion, fasting‐induced ketogenesis is reduced in HFHC diet‐fed H‐MDM2‐KO mice, but not in mice treated with Nutlin‐3a. The reduction in ketogenesis may be because of a secondary effect of increased TG‐VLDL secretion and/or direct regulation of the MDM2‐p53 axis on ketogenesis. We found that the change in ketogenesis in H‐MDM2‐KO mice was reversed by concomitant deletion of p53. Consistent with our finding, the MDM2‐p53 axis controls fatty acid oxidation under fasting conditions.^[^ ^16^ ^]^ Taken together, MDM2 plays a crucial role in the regulation of hepatic lipid metabolism in p53‐dependent and ‐independent manners under different nutritional status.

The severity of MAFLD was similar between DKO and WT littermates, although upregulation of ApoB expression and TG‐VLDL secretion were still observed in DKO mice. Further analysis showed that lipogenic enzymes including Acc1, Fasn, and Acly were augmented in the liver of DKO mice, which might neutralize the beneficial effect of increased TG‐VLDL secretion in DKO mice. These data suggest that hepatic p53 deficiency might contribute to an increased lipogenic response and subsequent steatosis. Indeed, hepatic‐specific downregulation of p53 leads to excessive lipid accumulation.^[^ ^26^, ^27^, ^28^ ^]^ Hepatic steatosis induced by p53 deficiency occurs by overactivation of p63 (the p53 ortholog) and subsequent activation of I*κ*B‐kinase *β* (IKK*β*), inflammation, ER stress, and lipogenesis.^[^ ^26^ ^]^ Adenovirus‐mediated overexpression of dominant‐negative p53 also results in hepatic steatosis, inflammation, and ER stress.^[^ ^27^ ^]^ In contrast, pharmacological activation of p53 by doxorubicin improves NASH, accompanied by downregulation of lipogenic enzymes and ER stress in the liver.^[^ ^27^ ^]^ p53 downregulation does not affect hepatic expression of ApoB100 and ApoB48proteins, whereas p53 activation reduces the lipogenic Fasn,^[^ ^26^, ^27^ ^]^ which is consistent with our findings in DKO mice.

In contrast to our findings, a previous study showed that genetic deletion of *MDM2* leads to hepatic fibrosis by the induction of p53‐mediated CTGF.^[^ ^44^ ^]^ The reason for this discrepancy is currently unknown but may be because of different housing environments, diets, and/or genetic backgrounds of the animals. In addition to the liver, inhibition of MDM2 by Nutlin‐3a exerts antifibrotic effects in hearts with myocardial infarction.^[^ ^45^ ^]^ Indeed, our present study employed both genetic and pharmacological approaches to show that inactivation of MDM2 not only prevents steatosis but also limits inflammation and fibrosis in rodent models with MAFLD. Increased ApoB and its associated TG‐VLDL secretion, but not p53 activation, are required for the hepatoprotective effects of Nutlin‐3a. However, further investigation on the hepatoprotective effect of Nutlin‐3a in mouse models with different severities of MAFLD is warranted.

In summary, we demonstrate that MDM2 inactivation by genetic and pharmacological approaches result in protective effects in two independent mouse models of MAFLD with steatosis, inflammation, and/or fibrosis. MDM2 is upregulated in the liver under obese conditions, and blocking its activity increases hepatic ApoB expression and TG‐VLDL secretion. Therefore, hepatic MDM2 and its ability to suppress ApoB expression are potential pharmacological targets for the treatment of MAFLD, a metabolic disease currently lacking any effective treatment.

## 4 Experimental Section

### Animal Studies

Hepatocyte‐specific *MDM2* knockout (H‐MDM2‐KO) mice were generated by crossing *MDM2* ^floxed/floxed^ mice (provided by Prof. Guillermina Lozano from the University of Texas M.D. Anderson Cancer Center) with albumin‐Cre transgenic mice (The Jackson Laboratory). The genotypes of H‐MDM2‐KO mice and their WT littermates are *MDM2* ^floxed/floxed^ *Alb‐Cre* ^+ve^ and *MDM2* ^floxed/floxed^, respectively. To generate hepatocyte‐specific *MDM2* and *p53* double knockout mice (DKO; *MDM2* ^floxed/floxed^ *p53* ^floxed/floxed^ *Alb‐Cre* ^+ve^) and their WT littermates (*MDM2* ^floxed/floxed^ *p53* ^floxed/floxed^), H‐MDM2‐KO (*MDM2* ^floxed/floxed^ *Alb‐Cre* ^+ve^) mice were crossed with *p53* ^floxed/floxed^ mice (The Jackson Laboratory). The *MDM2* ^floxed/floxed^ *p53* ^floxed/floxed^ WT littermates were used only in experiments involving DKO mice (Figure 6; and Figures S2E,F, S8, and S9, Supporting Information). All mice were maintained on the C57BL/6J genetic background. The animals were allocated to experimental groups according to their genotypes. The mice were fed STC (13% kcal fat, D5053, LabDiet, MO), HFHC diet (40% kcal with 0.15% cholesterol, D12079B, Research Diet, NJ), or CDAHFD (45% of calories from fat with 0.1% methionine, A06071309, Research Diet)^[^ ^31^ ^]^ from the age of 8 weeks. Leptin receptor‐deficient (*db*/*db)* mice and their lean controls (*db*/*+*) were obtained from the Jackson Laboratory. For pharmacological inhibition of MDM2, C57BL/6J mice were intraperitoneally injected with Nutlin‐3a (10 mg kg^−1^; catalog #S8059, Selleckchem, PA) or vehicle (DMSO in PBS) twice a day, as reported previously.^[^ ^15^ ^]^ For in vivo *ApoB* knockdown, mice were intravenously injected with Invivofectamine (catalog #IVF3001, Invitrogen) in complex with siRNA against *ApoB* (catalog #132 001, Assay ID#MSS215496, Invitrogen, MA) or Stealth siRNA negative control (catalog #100 002 301, Invitrogen) for 17 days, according to the manufacturer's instructions.

For *p53* knockdown in the liver, AAV expressing shRNA against mouse *p53* and *scramble control* under the control of miR30 promoter was generated by ViGene Biosciences Inc (Shandong, China). The shRNA sequences against mouse *p53* and *scramble control* are listed in Table S7 (Supporting Information). AAV particles were produced by cotransfection of AAV, pRep2Cap8, and helper vectors in HEK293 cells, followed by purification using ultracentrifugation and quantification using QPCR method. 12‐week‐old male C57BL/6J mice were injected with AAV‐shRNA against *p53* or *scramble control* through the tail vein at a dosage of 1 × 10^12^ genomic copy number per animal, and allowed for recovery and AAV expression for 4 weeks. The mice were then fed CDAHFD for 2 weeks, followed by treatment with Nutlin‐3a as described above.

Male mice were used in the experiments unless otherwise specified. The investigators were not blinded to the experimental groups. Mice were kept in a room with a 12 h light/dark cycle, humidity (60–70%), and temperature (23 ± 1 ℃) control. All mice had free access to food and water during experiments unless otherwise specified. All animal experiments were approved by the Animal Ethics Committee of the University of Hong Kong (3967‐14 and 3967‐16) and the Hong Kong Polytechnic University (17‐18/28‐HTI‐R‐HMRF and 19–20/55‐HTI‐R‐GRF).

### TG‐VLDL Secretion and TG Clearance Test

For TG‐VLDL secretion test, 16 h fasted mice were intravenously injected with tyloxapol (catalog #T8761, Sigma‐Aldrich, MO) at 500 mg kg^−1^ body weight. For TG clearance test, 16 h fasted mice were orally gavaged with olive oil at a dosage of 10 µL g^−1^ body weight. Serum TG levels were determined using Stanbio Triglycerides LiquiColor Test (catalog #2100, STANBIO Laboratory, TX), according to the manufacturer's instructions. Cholesterol and TG levels in different subclasses of lipoproteins were analyzed using proprietary gel‐filtration high‐performance liquid chromatography (LipoSEARCH) by Skylight Biotech Inc (Akita, Japan).

### Human Studies

Liver biopsy samples were collected from morbidly obese patients who underwent bariatric surgery at the First Affiliated Hospital of Jinan University, Guangzhou, China. The patients were recruited between July 2016 and February 2019. Patients who had the following conditions were excluded: heavy alcohol consumption (>210 g week^−1^ in men and >140 g week^−1^ in women), chronic hepatitis B or C, use of steatogenic drugs, other chronic liver diseases (including hemochromatosis, *α*1‐antitrypsin deficiency, and Wilson's disease), autoimmune liver disease, drug‐induced liver disease, liver cirrhosis‐related complications, hepatocarcinoma, and history of chronic inflammatory bowel disease. Circulating biomarkers related to liver injury (AST and ALT), lipid profiles [triglyceride, total cholesterol (TCHOL), lipoprotein LDL‐C, ApoB, and ApoA levels] were measured during routine blood tests. The clinical characteristics of human subjects are listed in Table S1 (Supporting Information). The study protocol conformed to the ethical guidelines of the Declaration of Helsinki Principles and was approved by the Institutional Review Board (IRB) of the Affiliated Hospital of Jinan University (Number: 2019–024). Written informed consents were obtained from all subjects.

The liver sections were subjected to H&E staining and Trichrome staining for the assessment of steatosis, inflammation, ballooning, and fibrosis. The histological analyses of the liver sections were independently conducted by two internationally renowned pathologists, Dr. Subrata Chakrabarti (Department of Pathology and Laboratory Medicine, Western University, Canada) and Dr. Sen Yan (Dr. Everett Chalmers Hospital, Fredericton, New Brunswick, Canada), in an unbiased and blinded manner. Histological assessment was conducted according to the histological scoring system developed by NASH Clinical Research Network^[^ ^46^ ^]^ as follows: i) steatosis: 0 (absence of steatosis), 1 (5–33%), 2 (34–66%), 3 (>66%); ii) infiltration of immune cell and lobular inflammation: 0 (none), 1 (one to two foci per 20X field), 2 (three‐four foci per 20X field), 3 (> four foci per 20X field); and iii) hepatocellular ballooning: 0 (absence of ballooned cell), 1 (few ballooned cells), 2 (many ballooned cells). NASH was defined as concomitant presence of steatosis, ballooning, and lobular inflammation with or without fibrosis.

Hepatic *MDM2* mRNA expression levels were compared in human with or without MAFLD using the transcriptome dataset GSE49541^[^ ^19^ ^]^ and Genome‐wide mRNA dataset from PMID: 21 737 566 (the Assession No.: E‐MEXP‐3291; http://www.webcitation.org/5zyoj Nu7T).^[^ ^20^ ^]^ The GSE49541 dataset identified 251 signature genes in 71 human NAFLD subjects with different degrees of fibrosis.^[^ ^19^ ^]^ Raw intensity expression file of GSE49541 was imported into R and subjected to normalization and background correction using Oligo package. The E‐MEXP‐3291 dataset containing 45 human subjects clinically defined normal, steatosis, and NASH was analyzed with Affymetrix GeneChip Human 1.0ST arrays.^[^ ^20^ ^]^ The generated array data was imported into Limma R packages to calculate the relative mRNA expression among the three groups. All values are presented in log_2_ scale.

### Histopathological Analysis

For H&E staining, the liver samples were fixed in 4% formaldehyde in PBS for 1 day, followed by tissue processing, cut into 5 µm sections, deparaffinization with xylene, and washed with serial dilutions of ethanol. The sectioned slides were stained for nuclei with hematoxylin solution (ThermoFisher Scientific, MA) for 30 s, washed with acid alcohol for 1 s, stained with eosin for 3 s, washed with 100% ethanol for twice, and then cleared with xylene for 10 min and finally mounted with DPX mounting medium (ThermoFisher Scientific). For Picro Sirius staining, the sectioned slides were stained with hematoxylin solution for 50 s and then washed with acid alcohol for 1 s. Tissue slides were incubated with 0.1% Picro Sirius Red (catalog #ab150681, Abcam, Cambridge, UK) in picric acid for 1 h, washed with fresh acidified water (0.5% acetic acid glacial) for twice and dehydrated with 100% ethanol for three times. Tissue slides were cleared with xylene for 10 min and mounted with DPX mounting medium.

For the immunohistochemistry staining of Ki67, mouse liver sections were immersed into an antigen retrieval buffer (10 × 10^−3^ m sodium citrate, 0.05% Tween 20, pH 6.0) at 60 °C for 20 min, and then cooled down to room temperature. Next, the slides were incubated with 3% hydrogen peroxide (H_2_O_2_) for 15 min at room temperature in the dark for quenching of internal peroxidases. The slides were incubated with blocking buffer [10% FBS, 3% BSA in 1 X PBST (0.05% Tween 20 in 1 X PBS)] at room temperature for 1 h, followed by incubation with Ki67 antibody (catalog#ab15580, Abcam, 1:200) at 4 °C overnight. The slides were then washed with 1 X PBST four times, incubated with secondary antibody conjugated with HRP (1:500) at room temperature for 1 h, and washed with 1 X PBST, followed by incubation with 3,3'‐diaminobenzidine (DAB) solution. The slides were counterstained with hematoxylin and mounted with DPX mounting medium. Images were captured using a microscopy (BX35‐DP80, Olympus) and analyzed using ImageJ.

For the immunostaining of MDM2, human liver sections were subjected to antigen retrieval using a Tris‐EDTA Buffer (10 × 10^−3^ m Tris Base, 1 × 10^−3^ m EDTA Solution, 0.01% Triton‐X 100, pH 9.0) at 60 °C for 20 min. The sections were then treated with 3% H_2_O_2_ for 10 min and incubated with a blocking buffer (3% FBS, 3% BSA, and 0.01% Tween 20 in 1 X PBS) at room temperature for 1 h, followed by incubation with the MDM2 antibody (catalog#OP‐115, EMD Millipore, 1:100; USA) at 4 °C overnight. The slides were washed, incubated with the secondary antibody, developed with DAB, counterstained, mounted, and analyzed as above.

### Hepatic TG Content

Liver tissues (≈50 mg) were homogenized with 200 µL PBS, followed by mixing with 5 mL chloroform‐methanol mixture (chloroform: methanol, 2:1, v/v, with 151 × 10^−6^ m butylated hydroxytoluene) at 4 °C overnight. An aliquot of 1 mL of the mixture was washed with 200 µL of saline, followed by centrifugation at 5000 g for 15 min. The chloroform layer was collected and air‐dried, and then resuspended in 100% ethanol. The TG levels were measured using the TG assay kit and normalized with protein concentration of tissue lysates.

### Biochemical and Immunological Analysis

Blood glucose levels were determined using a glucometer (ACCU‐Check glucose meter, Roche, MO). Serum cholesterol and FFA levels were measured using Cholesterol Liquicolor (Catalog #1010, STANBIO Laboratory) and Half Micro Test Kit (Catalog #11 383 175 001, Roche), respectively. Serum MDA level was determined using OxiSelect TBARS Assay Kit (Catalog #STA‐330, CELL BIOLABS, CA). Serum Mcp‐1 protein level was measured by a Mcp‐1 ELISA kit (Catalog #DY479‐05, R&D, MN). Serum levels of ALT and AST were measured using ALT/GPT Liqui‐UV kit (Catalog #2930/430, STANBIO Laboratory) and AST/SGOT Liqui‐UV kit (Catalog #2930/2920, STANBIO Laboratory), respectively.

### Cell Culture and Transfection

HepG2 cells were obtained from American Type Culture Collection (ATCC) and cultured in DMEM (catalog #12 800 082, ThermoFisher Scientific) supplemented with 10% fetal bovine serum (Catalog #10 270, ThermoFisher Scientific) and 1% penicillin and streptomycin (Catalog #15 140 122, ThermoFisher Scientific). HepG2 cells were cotransfected with siRNA against *MDM2* or *scramble* and/or *p53*, or plasmids encoding *MDM2* or its E3 ligase defective mutants and/or HA‐ubiquitin or its mutant using Lipofectamine 3000 (Catalog #L300015, ThermoFisher Scientific) according to manufacturer's instruction. Secretions of ApoB and albumin in conditional medium were measured by ApoB (Catalog #3715‐1H‐6, Mabtech, Sweden) and albumin (Catalog #E80‐129A, Bethyl Laboratories Inc, TX) ELISA kits according to manufacturer's manuals.

To access cell viability, transfected or Nutlin‐3a treated HepG2 cells were incubated with medium containing 0.25 mg mL^−1^ 3‐(4,5‐dimethylthiazol‐2‐yl)‐2,5‐diphenyltetrazolium bromide (MTT, Catalog #M1025, Solarbio Life Sciences, Beijing, China) at a 37 °C CO_2_ incubator for 4 h. Medium was removed and produced purple crystals were solubilized with 100 µL of DMSO, followed by shaking for 15 min and measurement of optical density at 570 nm.

To isolate primary hepatocytes from C57BL/6J mice or H‐MDM2‐KO mice and their WT littermates fed the HFHC diet, liver was perfused with a pre‐warmed perfusion buffer (119.78 × 10^−3^ m NaCl, 23.8 × 10^−3^ m NaHCO3, 5 × 10^−3^ m HEPES, 4.8 × 10^−3^ m KCl, 1.2 × 10^−3^ m MgSO4, 1.2 × 10^−3^ m KH_2_PO_4_, and 5.55 × 10^−3^ m glucose, pH 7.4, 1% penicillin‐streptomycin) containing 1 × 10^−3^ m EGTA to wash out the blood, followed by digestion with perfusion buffer containing 0.5 mg mL^−1^ collagenase IV and 5 × 10^−3^ m CaCl_2_. The digested liver was gently dissociated in cold William's E medium (Catalog #12 551 032, Gibco, MA) supplemented with 10% FBS, 1% penicillin‐streptomycin and 2 × 10^−3^ m L‐glutamine (Catalog #25 030 081, Gibco) (This medium is so‐called complete William's E medium). The cell suspension was transferred to a 100 µm Falcon strainer (Catalog #352 360, Corning Incorporated, NY) and washed with William's E medium by centrifugation at 50 × g for 3 min at 4 °C, followed by resuspension with complete William's E medium. 5 × 10^5^ hepatocytes were plated onto 6‐well‐plates, cultured at 37 °C for 4 h, changed with fresh complete William's E medium and incubated for 20 h. The cells were treated with oleic acid (OA) conjugated with 5% fatty acid‐free BSA or BSA as control for 8 h. Secretion of ApoB in the conditional media was measured by mouse ApoB ELISA Kit (Catalog #E‐EL‐M3017, Elabscience Biotechnology Inc, Wuhan, China) according to manufacturer's manual and normalized with cell protein concentration.

### BODIPY Lipid Staining

Primary hepatocytes or HepG2 cells were washed twice with PBS and then cultured with William's E medium or DMEM containing 1% fatty acid‐free bovine serum albumin (BSA, Catalog # A8806, Sigma‐Aldrich), respectively. The cells with or without OA treatment were rinsed twice with PBS and incubated with BODIPY 493/503 lipid probe (5 × 10^−6^ m; Catalog # D3922, Invitrogen) in PBS in the dark at 37 °C for 10 min, followed by fixation with 10% neutral formalin at room temperature for 30 min. The fixed cells were rinsed with PBS three times. The intracellular fluorescent signals were captured either by Nikon Eclipse Ni‐U Fluorescent Microscope (Figure 4C; and Figures S6D and S12C, Supporting Information) or Thermo Scientific Varioskan LUX Multimode Microplate Reader (Figure 4I). The BODIPY florescent intensity captured by Fluorescent Microscope was quantified by Image J and normalized with the cell size.

### Generation of Truncated ApoB Expression Vectors and Their Mutants with Defective MDM2 Binding Ability

The vectors expressing ApoB fragments that contain putative MDM2 binding motifs were generated by amplifying the human ApoB coding region spanning 1–1872 and 10 257–12 054 bp (1 is defined as the start codon) (Shanghai Genechem Co., LTD, China) (Figure S11B, Supporting Information). The above regions encode amino acid residues 1–624 and 3419–4018 of ApoB, respectively. The putative binding motifs 345–354 amino acids and 3682–3691 amino acids were mutated as follows: L347/F348/L351A or L3684/W3685/L3688A, respectively. The sequences of constructs were confirmed by DNA sequencing and their expressions were confirmed by immunoblotting analysis.

### Coimmunoprecipitation

Liver tissues, HepG2 cells or primary hepatocytes were solubilized in a coimmunoprecipitation lysis buffer (50 × 10^−3^ m Tris‐HCl, pH 7.5, 250 × 10^−3^ m NaCl, 1% Nonidet P [NP]‐40, 1 × 10^−3^ m EDTA), supplemented with 1 × 10^−3^ m DTT and protease inhibitor cocktail (Catalog #4 693 159 001, Roche). Total lysates (500 mg) were incubated with ApoB antibody (Catalog #20 737, Abcam), Myc antibody (Catalog #sc‐40, Santa Cruz Biotechnology, TX) or rabbit IgG at 4 °C overnight. The immunocomplexes were incubated with Protein A agarose at 4 °C for 1 h, washed with cold coimmunoprecipitation lysis buffer three times, and subjected to immunoblotting analysis of MDM2. The effects of oleic acid (0.4 × 10^−3^ m), stearic acid (0.4 × 10^−3^ m), and Nutlin‐3a (10 × 10^−6^ m) on ApoB‐MDM2 interaction were tested in HepG2 cells by coimmunoprecipitation assay as described above. For immunoprecipitation of FLAG‐tagged ApoB, total cell lysates were incubated with agarose beads conjugated with anti‐FLAG antibody (catalog #M8823, Sigma‐Aldrich) at 4 °C for 2 h. The immunocomplexes and total cell lysates were subjected to immunoblotting analysis of FLAG (Catalog #A8592, Sigma‐Aldrich, 1:2000) and Myc (catalog #sc‐40, Santa Cruz Biotechnology, 1:1000).

### Real‐Time Quantitative PCR Analysis

Total RNA was extracted with Trizol (Catalog #15 596 026, Invitrogen). 500 ng RNA was used for cDNA synthesis using GoScript (Catalog #A2801, Promega, WI) or PrimeScript RT reagent Kit (Catalog #RR037A, Takara, Shiga, Japan). Real‐time PCR analysis was performed using QuantiNova SYBR Green RT‐PCR Kit (Catalog #208 154, Qiagen, MD) on Applied Biosystems ViiA 7 Real‐Time PCR System (Applied Biosystems, CA). Gene expression level was analyzed using the ∆∆Ct threshold cycle method and normalized against the *18s RNA*, *GAPDH*, *36B4*, or *b‐actin* as indicated in figure legends. Primer sequences are shown in Table S8 (Supporting Information).

### Measurement of Caspase 3 Activity

To assess cell apoptosis, caspase 3 activity was measured using the EnzChek Caspase‐3 Assay Kit, Z‐DEVD‐AMC substrate (Catalog # E13183, ThermoFisher Scientific) according to the manufacturer's instructions. Briefly, the cells were washed with PBS and lysed with cell lysis buffer from the kit and then centrifuged at 5000 rpm for 5 min. 50 µL of cell lysate (50 µg of total protein) was mixed with 50 µL of 2 X substrate working solution, followed by incubation at room temperature for 30 min and measurement of fluorescent density at excitation/emission of 342/441 using Thermo Scientific Varioskan LUX Multimode Microplate Reader. The caspase 3 activity was normalized with protein concentration of the lysate.

### Immunoblotting Analysis

Protein samples from liver tissues and cultured cells were extracted with RIPA lysis buffer (65 × 10^−3^ m Tris‐HCl, pH 7.5, 150 × 10^−3^ m NaCl, 1 × 10^−3^ m EDTA, 1% Nonidet P‐40, 0.5% sodium deoxycholate, 1% SDS), supplemented with 1 × 10^−6^ m DTT and protease inhibitors mixture (Catalog # 4 693 159 001, Roche). Protein samples were resolved by sodium dodecyl sulfate polyacrylamide gels (SDS‐PAGE) and separated proteins were electro‐transblotted onto polyvinylidene fluoride membrane (Catalog #45 004 110, GE Healthcare, UT). For detection of ApoB in liver tissues or cultured cells, protein was transferred using a modified transfer buffer (40 × 10^−3^ m Tris, 20 × 10^−3^ m sodium acetate, 2 × 10^−3^ m EDTA, pH 7.4, 10% v/v methanol, 0.005% w/v SDS) at 350 mA for 6 h at 4 °C. Primary antibodies against MDM2 (Catalog #OP115‐100, EMD MILLIPORE, 1:2500), p53 (Catalog #2524S, Cell Signaling Technology, 1:1000; MA), p21 (Catalog #ab7960, Abcam, 1:2500), HSP90 (Catalog #4874S, Cell Signaling Technology, 1:2500), β‐tubulin (Catalog #ab15568, Abcam, 1:2500), ApoB for liver (Catalog # ab20737, Abcam, 1:2500), ApoB for cell and serum (Catalog #sc‐11795, Santa Cruz Biotechnology, 1:1000), MTTP (Catalog #612 022, BD Transduction Laboratories, 1: 5000), ApoA‐1 (Catalog #ab33470, Abcam, 1:2500), PCB (Catalog #128 952, Abcam, 1:2500), Albumin (Catalog #66051‐1‐Ig, Proteintech, 1:5000; Wuhan, China), HA tag (catalog #2999S, Cell Signaling Technology, 1:5000), GAPDH (Catalog #5174S, Cell Signaling Technology, 1:2500), ACC1 antibody (Catalog # 3676P, Cell Signaling Technology, 1:1000), FASN antibody (Catalog #3189S, Cell Signaling Technology, 1:1000), p21 antibody (Catalog # ab7960, Abcam, 1:2000), Myc antibody (Catalog #sc‐40, Santa Cruz Biotechnology,1:1000) and FLAG antibody (Catalog #A8592, Sigma‐Aldrich, 1:2000), and antimouse secondary antibody (Catalog #7076S, Cell Signaling Technology, 1:2500), anti‐rabbit secondary antibody (Catalog #7074S, Cell Signaling Technology, 1:2500) and antigoat secondary antibody (Catalog #705‐035‐147, Jackson ImmunoResearch, 1:5000; PA) were used.

### Measurement of Hepatic Oxidative Stress

Dihydroethidium (DHE) staining was performed to visualize the formation of reactive oxygen species. Frozen liver sections were fixed in 4% paraformaldehyde for 5 min, and then washed with PBST (PBS supplied with 0.1% Tween 20) three times. DHE solution (Catalog #sc‐2/4894, Santa Cruz Biotechnology) was added to the samples and incubated at 37 °C for 30 min in the dark, followed by washing with PBST three times. Fluorescent images were immediately captured using a fluorescent microscope (Nikon Eclipse Ni, Japan) at 200 X magnification. Malondialdehyde (MDA) level in the liver was measured using OxiSelect TBARS Assay Kit (Catalog #STA‐330, CELL BIOLABS) according to manufacturer's manual. The concentration of MDA was normalized with protein concentration of tissue lysates.

### Detection of ApoB Ubiquitination

HepG2 cells with ectopic expression of HA‐tagged ubiquitin and Myc‐tagged MDM2 were lysed with a ubiquitination lysis buffer (2% SDS, 150 × 10^−3^ m NaCl, 10 × 10^−3^ m Tris‐HCl, 2 × 10^−3^ m sodium orthovanadate, pH 8.0, 10 × 10^−3^ m *N*‐ethylmaleimide, 1 × 10^−3^ m dithiothreitol [DTT] and protease inhibitors cocktail [Catalog #4 693 159 001, Roche]). The cell lysates were boiled for 10 min, followed by dilution with an immunoprecipitation buffer (10 × 10^−3^ m Tris‐HCl, pH 8.0, 150 × 10^−3^ m NaCl, 2 × 10^−3^ m EDTA, 1% Triton‐X 100) at 1:2 ratio. The diluted lysate was centrifuged at 20 000g for 30 min. 500 µg of total cell lysate was mixed with the immunoprecipitation buffer to a final volume of 250 µL, followed by incubation with 5 µL polyclonal antibody against ApoB (Catalog #ab20737, Abcam) at 4 °C overnight. The immunocomplex was captured by Protein A agarose (Catalog #SC2001, Santa Cruz Biotechnology) at 4 °C for 1 h, followed by washing with cold immunoprecipitation buffer three times. The immunocomplex was eluted by boiling at 99 °C for 10 min and subjected to immunoblotting analysis as specified in each figure legend.

For the in vitro ubiquitination of ApoB, recombinant human ubiquitin E1 enzyme (50 ng; Catalog #E‐304‐050, R&D), recombinant human E2 enzyme (200 ng; Catalog #K‐980B, R&D), recombinant human MDM2 protein (250 ng; Catalog #E3‐204‐050, R&D), native ApoB protein from human plasma (250 ng; Catalog #LS‐G5072‐500, Lifespan, RI), and recombinant human HA‐Ub protein (5 µg; Catalog #U‐110‐01M, R&D) were mixed in a reaction buffer (50 × 10^−3^ m Tris pH 7.5, 2 × 10^−3^ m ATP, 5 × 10^−3^ m MgCl_2_, 2 × 10^−3^ m DTT). The reaction mixture was incubated at 30 ℃ for 1.5 h, and then stopped by adding 4 X SDS sample buffer (0.2 m Tris, pH 6.8, 8% SDS, 40% glycerol, 0.004% bromophenol blue). The reaction mixture was boiled at 100 ℃ for 5 min, followed by immunoprecipitation of ApoB using anti‐ApoB antibody (Catalog# sc‐393636 Santa Cruz Biotechnology, 1:100). The immunocomplex was subjected to immunoblotting analysis using anti‐HA antibody (Catalog #3724S, Cell Signaling Technology, 1:2500) and ApoB (Catalog #sc‐393636, Santa Cruz Biotechnology, 1:2000).

### In Vitro Lipid Secretion Assay

HepG2 cells were labeled with [^3^H] glycerol (2 mCi mL^–1^, PerkinElmer, UT) in the presence of oleic acid (0.4 × 10^−3^ m) for 8 h. Radioactivity of lipid fraction extracted from the conditional medium was measured by a liquid scintillation counter.

### Materials and Reagents

Cycloheximide (Catalog #0 1810), MG132 (Catalog #M8699), collagenase IV (Catalog #C5138), EGTA (Catalog #324 626), oleic acid (Catalog #O1008), and fatty‐acid free BSA (Catalog # A8806) were purchased from Sigma‐Aldrich. Plasmids encoding HA‐ubiquitin and its mutants were gifts from Dr. Edward Yeh (University of Texas M.D. Anderson Cancer Center). pcDNA3‐Myc‐MDM2 was a generous gift from Dr. Jean‐Christophe Marine.

### Statistical Analysis

The data were expressed as mean ± standard error of mean. All statistical analyses were performed using Prism 9 (GraphPad Software) or SPSS. All the data are not pre‐processed, except the hepatic MDM2 expression in humans are presented as log2 scale in Figure 1G,H; and Figure S4 (Supporting Information). Sample size (*n*) of each experiment is listed in the figure legends. When parametric tests were used, all the data were pretested for normality and equal variance among the groups using D'Agostino‐Pearson omnibus normality test and Levene's test, respectively. Statistical significance of normally distributed data with equal variance was analyzed using two‐tailed Student's *t*‐test or One‐way ANOVA with Tukey correction for multiple comparisons as indicated in the figure legends. Nonparametric tests, including Mann Whitney u test, Welch's *T*‐test, Welch's ANOVA followed by Dunnett's test, and Kruskal Wallis test followed by Dunn's test were used, when the data do not normally distribute and/or have unequal variance among the groups. Spearman correlation analysis was used to examine the association between hepatic MDM2 expression and circulating ApoB levels in Figure S4 (Supporting Information). Two‐tailed tests are used in the data analysis unless otherwise specified. A *p* value of *p* < 0.05 was considered as statistical significance. Representation of the *p*‐value was *p < 0.05, **p < 0.01 or specified in the figure legend. Animal experiments were conducted one time or two times with each animal as a biologically independent sample. All experiments were repeated at least three times.

## Conflict of Interest

The authors declare no conflict of interest.

## Author Contributions

A.X., K.K.Y.C., H.L., L.W., and Z.L. contributed equally to this work. H.L. and L.W. performed most of the experiments and drafted the manuscript. Z.L. generated some of the data and drafted the manuscript. K.L., M.K., D.Y., X.C, K.W., K.K.L.W., and M.F. performed the experiments. C.W. and E.S. are responsible for the supervision in collection of liver samples from obese subjects underwent the bariatric surgery. P.H. initiated the project and contributed to study design. H.X. and R.L.C.H. designed the experiments and edited the manuscript. H.P. contributed to experimental design and data analysis. A.X. and K.K.Y.C. contributed to experimental design, supervision of the work, funding acquisition, and manuscript writing.

## Supporting information

Supporting Information

## Data Availability

The data that support the findings of this study are available from the corresponding author upon reasonable request.
