# Supplementary material for: Hepatic MDM2 Causes Metabolic Associated Fatty Liver Disease by Blocking Triglyceride‐VLDL Secretion via ApoB Degradation
Source: Adv Sci (Weinh). 2022 May 7;9(20):2200742. doi: 10.1002/advs.202200742 (PMC9284139; doi:10.1002/advs.202200742)
Supplement: Supplementary file 1 — Supporting Information [file ADVS-9-2200742-s001.pdf]

ADVANCED SCIENCE
Open Access

Supporting Information

for *Adv. Sci.*, DOI 10.1002/advs.202200742

Hepatic MDM2 Causes Metabolic Associated Fatty Liver Disease by Blocking Triglyceride-VLDL Secretion via ApoB Degradation

Huige Lin, Lin Wang, Zhuohao Liu, Kekao Long, Mengjie Kong, Dewei Ye, Xi Chen, Kai Wang, Kelvin KL Wu, Mengqi Fan, Erfei Song, Cunchuan Wang, Ruby LC Hoo, Xiaoyan Hui, Philip Hallenborg, Hailong Piao, Aimin Xu and Kenneth KY Cheng**

Supporting Information

Hepatic MDM2 Causes Metabolic Associated Fatty Liver Disease by Blocking Triglyceride-VLDL Secretion via ApoB Degradation

Huige Lin[#], Lin Wang[#], Zhuohao Liu[#], Kekao Long, Mengjie Kong, Dewei Ye, Xi Chen, Kai Wang, Kelvin KL Wu, Mengqi Fan, Erfei Song, Cunchuan Wang, Ruby LC Hoo, Xiaoyan Hui, Philip Hallenborg, Hailong Piao, Aimin Xu, Kenneth KY Cheng**

* or # These authors contributed equally.

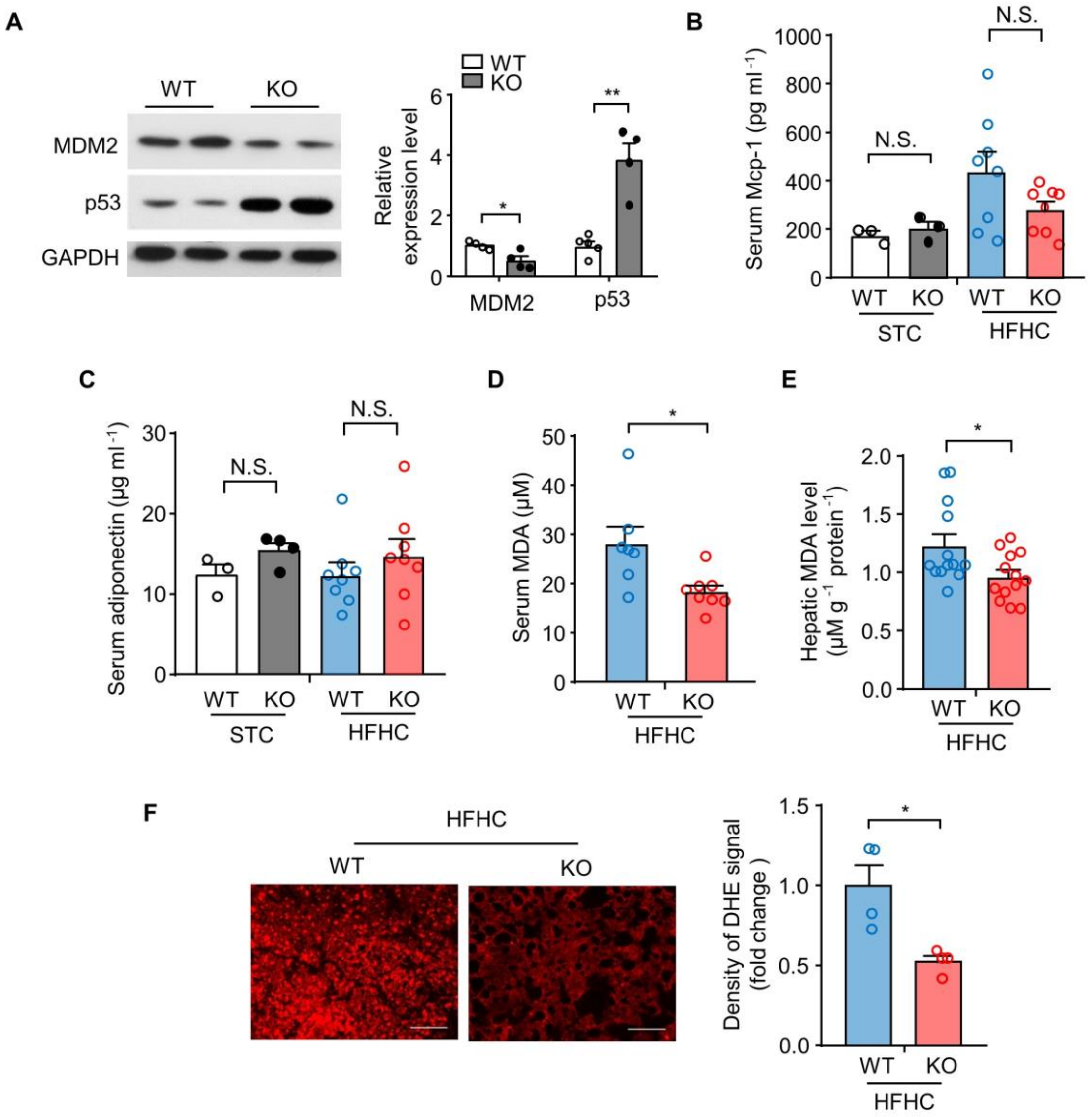

Figure S1. Hepatic deletion of MDM2 alleviates HFHC diet-induced systematic inflammation and hepatic oxidative stress. (A) Liver samples from 8-week-old H-MDM2-KO and their WT littermates were subjected to immunoblotting analysis of MDM2, p53 and GAPDH. The bar chart is densitometric analysis of MDM2 and p53 normalized with GAPDH (n=4-5). (B-F) H-MDM2-KO and WT littermates fed STC or HFHC diet for 16 weeks were used. Serum levels of (B) Mcp-1 and (C) adiponectin (n=3-8). (D) Serum levels of MDA (n=7-8). (E) Hepatic levels of MDA normalized with protein concentration (n=13). (F) Dihydroethidium (DHE) staining for detection of reactive oxidative species (ROS) formation (red signal) in liver sections. Scale bar: 100 µm. The right panel is quantification of intensities of ROS positive signal expressed as fold change over WT controls (n=4). All data are presented as mean ± SEM. *$p < 0.05$. (U test for panel D and two-tailed independent Student's t test for remaining data). Not statistical significance (N.S)

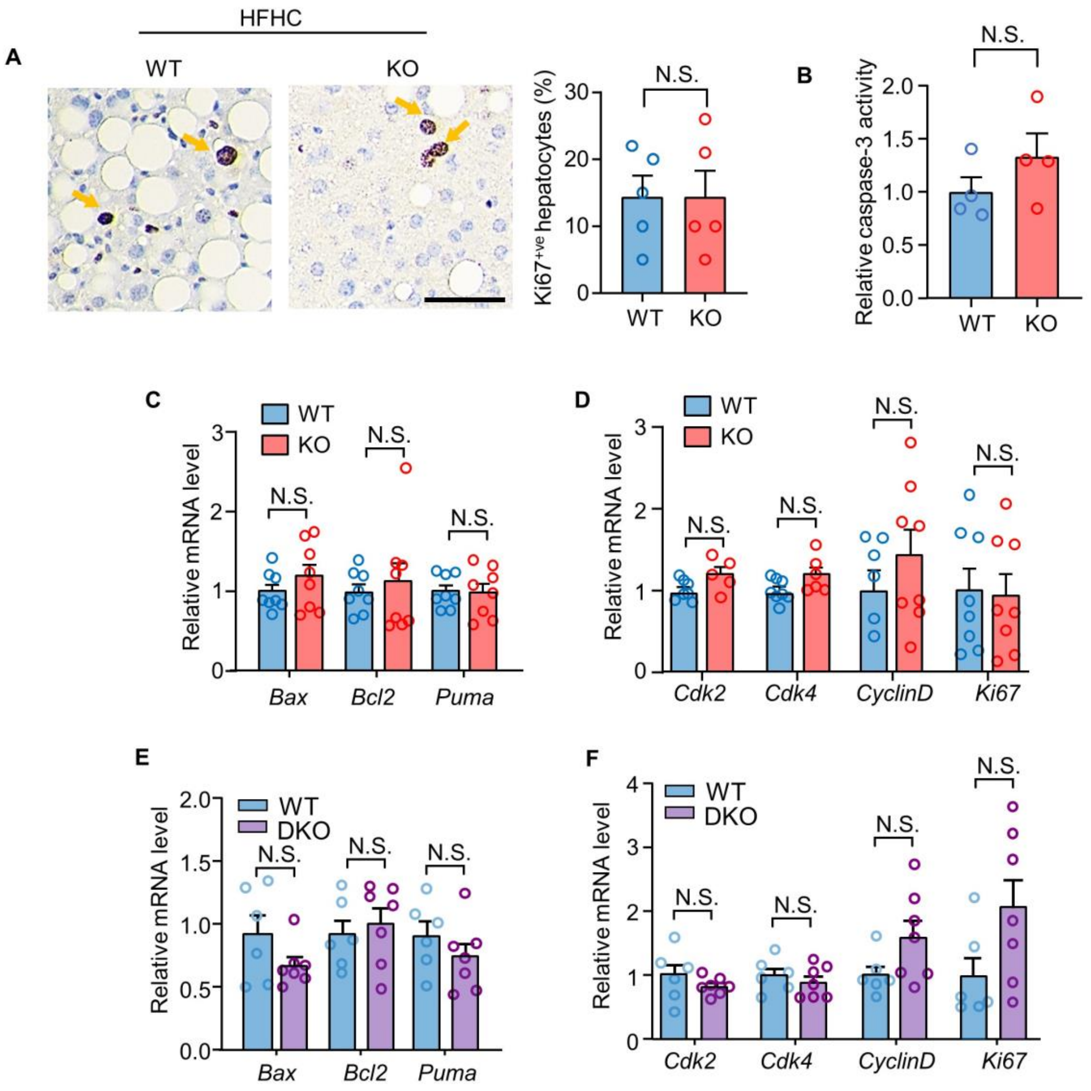

Figure S2. Cell apoptosis and proliferation in livers of H-MDM2-KO, H-MDM2-p53 double KO and their WT littermates. (A-D) The livers were collected from 24-week-old male H-MDM2-KO and their WT littermates fed HFHC diet for 16 weeks for the following analyses. (A) Ki67 staining of liver sections. Yellow arrows indicate Ki67 positive hepatocytes. Scale bar: 50 μm (n=5). (B) Activity of caspase-3 in liver lysates normalized with protein concentration (n=4). (C-D) mRNA levels of p53-regulated genes related with cell apoptosis (panel C: B-cell lymphoma 2 [*Bcl2*], BCL2-associated X protein [*Bax*], and the p53 upregulated modulator of apoptosis [*Puma*]), and cell proliferation (panel D: cyclin-dependent kinase 2 [*Cdk2*], *Cdk4*, *Cyclin D* and *Ki67*) (n=6-8). (E-F) Livers from 24-week-old HFHC-fed DKO and their WT littermates were subjected to QPCR analysis of genes related to (E) cell apoptosis and (F) cell proliferation. The target genes were normalized with *18s* and are expressed as fold change over WT (n=6-7). All data are presented as mean ±SEM. (U test for *Bcl2* in panel C; Welch's t-test for *Bax* in panel E; two-tailed independent Student's t test for the remaining data). Not statistical significance (N.S.)

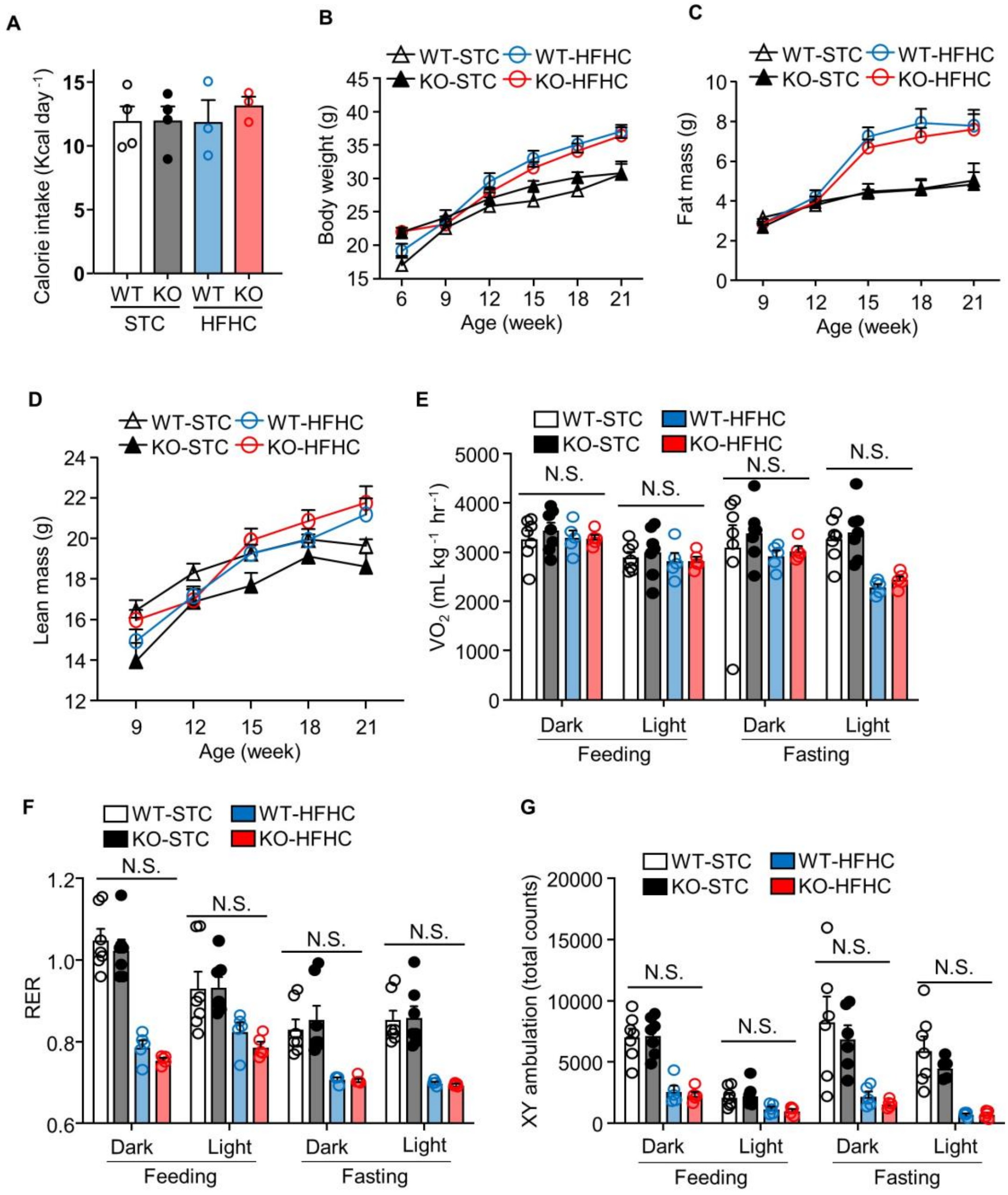

Figure S3. Effect of hepatic deletion of MDM2 on energy metabolism. 8-week-old H-MDM2-KO and their WT littermates were fed STC or HFHC diet for 16 weeks. (A) Daily calorie intake at the age of week-18 (n=4 for STC, and n=3 for HFHC groups). (B) Body weight, (C) fat mass, and (D) lean mass were measured every 3 weeks (n=5 for STC, n=9 for HFHC groups). (E-G) 16-week-old H-MDM2-KO and WT littermates fed HFHC diet or STC were monitored in a comprehensive animal monitoring system under *ad libitum* feeding and 24-hour fasting conditions. (E) Oxygen consumption, (F) Respiratory exchange ratio (RER), and (G) locomotor activity (n=7 for STC, n=5 for HFHC groups). All data are presented as mean ± SEM. (Two-tailed independent Student's t test). Not statistical significance (N.S)

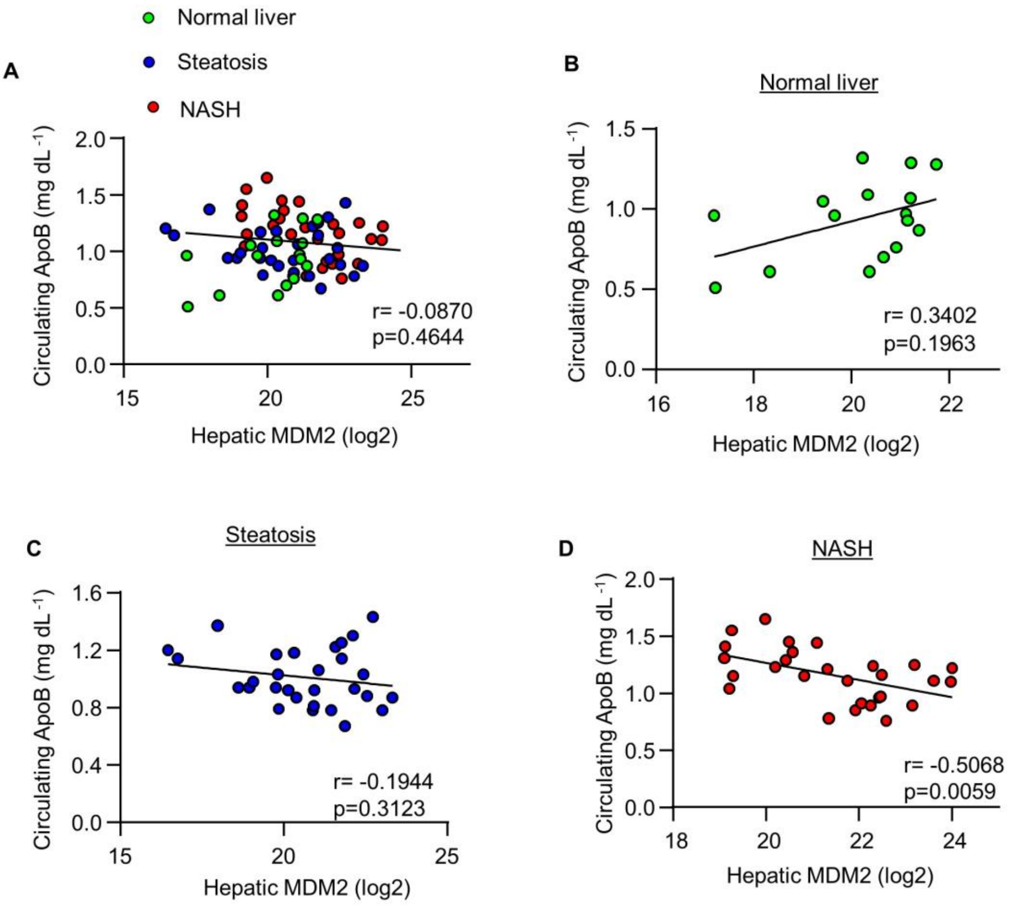

Figure S4. Correlation analysis of hepatic MDM2 expression with circulating ApoB levels in human obese subjects with or without MAFLD. Clinical characteristics of human subjects were described in Table S1. Hepatic expression of MDM2 was determined by immunohistochemical staining as shown in Figure 1E. Spearman correlation analysis between hepatic MDM2 expression and circulating ApoB levels in (A) all subjects (n=76), (B) normal liver group (n=19), (C) steatosis group (n=29), and (D) NASH group (n=28) are shown.

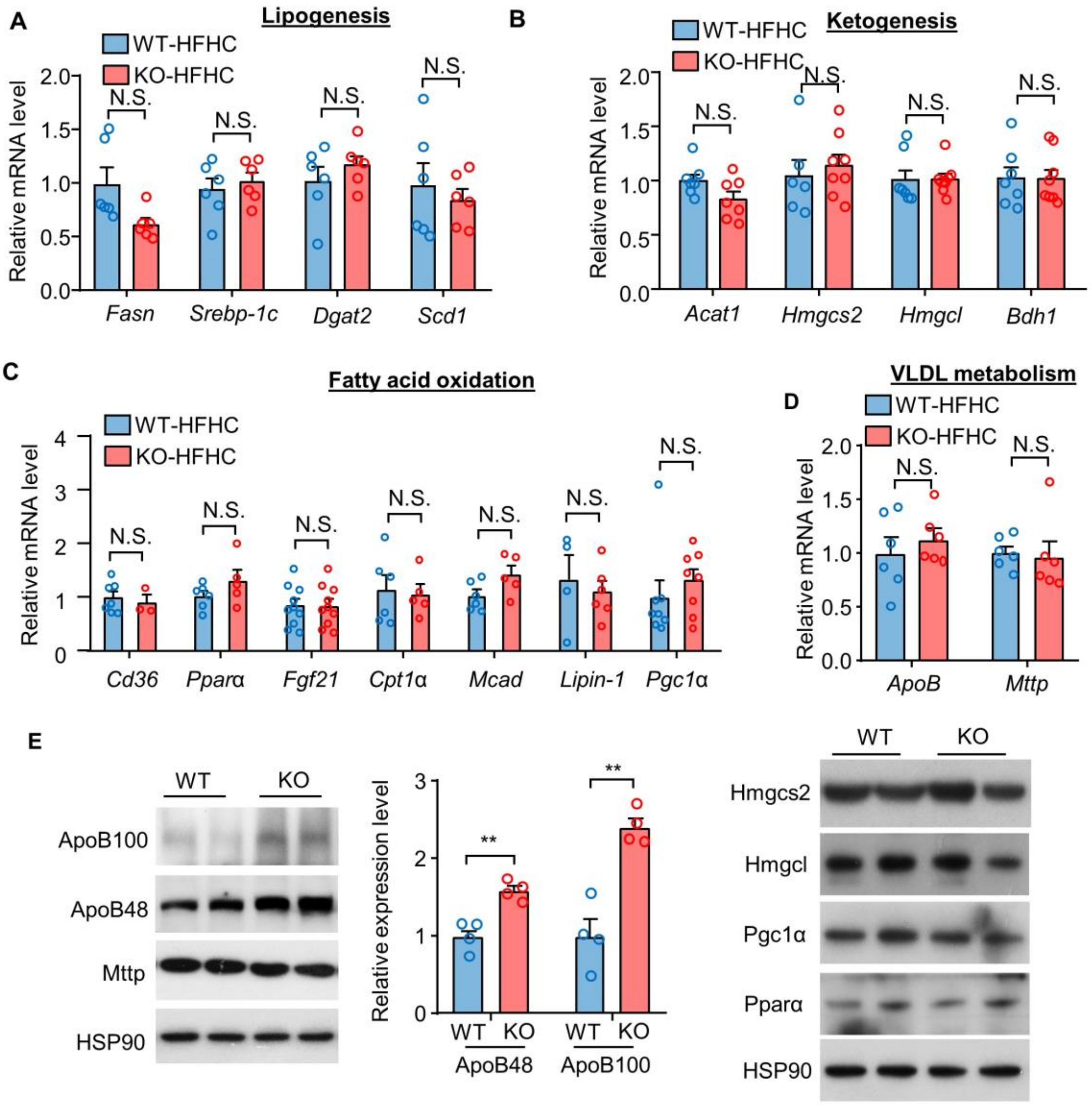

Figure S5. Effect of MDM2 deficiency on genes and proteins involved in lipid metabolism. Liver samples from H-MDM2-KO and their WT littermates fed HFHC diet for 16 weeks were used. Relative mRNA expressions of genes involved in (A) lipogenesis (*Fasn*, *Srebp-1c*, *Dgat2*, *and Scd1*); (B) ketogenesis (*Acat1*, *Hmgcs2*, *Hmgcl*, and *Bdh1*); (C) fatty acid oxidation (*Cd36*, *Pparα*, *Fgf21*, *Cpt1α*, *Mcad, Lipin-1*, *and Pgc1α*); (D) VLDL metabolism (*ApoB* and *Mttp*) (n=5-8). (E) Representative immunoblots of ApoB100, ApoB48, Mttp, Hmgcs2, Hmgcl, Pgc1α, Pparα and HSP90. The bar chart is densitometric analysis for the relative abundance of ApoB48 and ApoB100 normalized with HSP90 (n=4). All data are presented as mean ± SEM. *p<0.05 and **p<0.01. (U test for *Mttp* in panel D and two-tailed independent Student's t test for the remaining data). Not statistical significance (N.S)

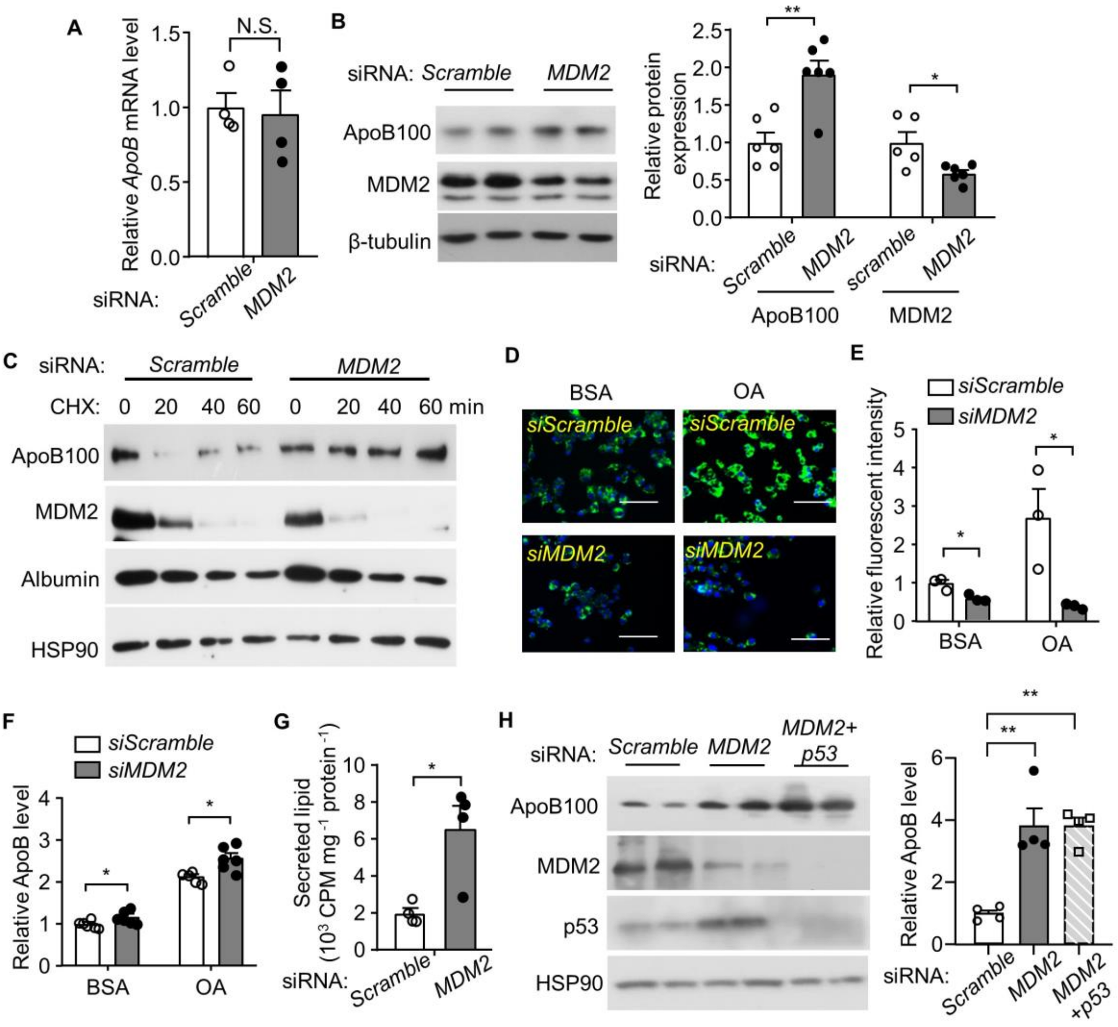

Figure S6. MDM2 deficiency increases ApoB protein expression by preventing its degradation in hepatocytes through a p53-independent manner. (A-G) HepG2 cells were transfected with siRNA against *MDM2* or *Scramble* for 48 hours. (A) mRNA levels of *ApoB* normalized with *β-tubulin* (n=4). (B) Immunoblotting analysis of ApoB100, MDM2, and β-tubulin in the transfected cells. The bar chart is the relative expressions of ApoB and MDM2 normalized with β-tubulin (n=5-6). (C) The transfected cells were treated with CHX (50 μg ml^{-1}) for different time points, followed by immunoblotting analysis as indicated. (D-F) The transfected cells were incubated with 0.4 mM OA conjugated with BSA or BSA for 8 hours. (D) The cells were fixed and stained with BODIPY lipid probes (green) and DAPI (blue). (scale bar: 100 μm) (E) The relative intensities of green over blue fluorescence (n=3). (F) The relative ApoB100 levels in culture medium normalized with albumin. There was no difference in albumin secretion among the groups (n=5-6). (G) The transfected cells were labelled with [^{3}H] glycerol in the presence of OA (0.4 mM) for 8 hours. Radioactivity of secreted lipid in conditional medium was measured and normalized with total protein (n=4). (H) HepG2 cells were transfected with siRNA against *MDM2*, *p53* or *Scramble* for 48 hours and then treated with 0.4 mM OA for 8 hours, followed by immunoblotting analysis as indicated. The bar chart is the relative expressions of ApoB normalized with HSP90 (n=4). Representative images are shown. All data are presented as mean ± SEM. *$p< 0.05$ and **$p<0.01$. (U test for panel G; One-way-ANOVA with Tukey test for panel H and two-tailed independent Student's t test for the remaining data). Not statistical significance (N.S.)

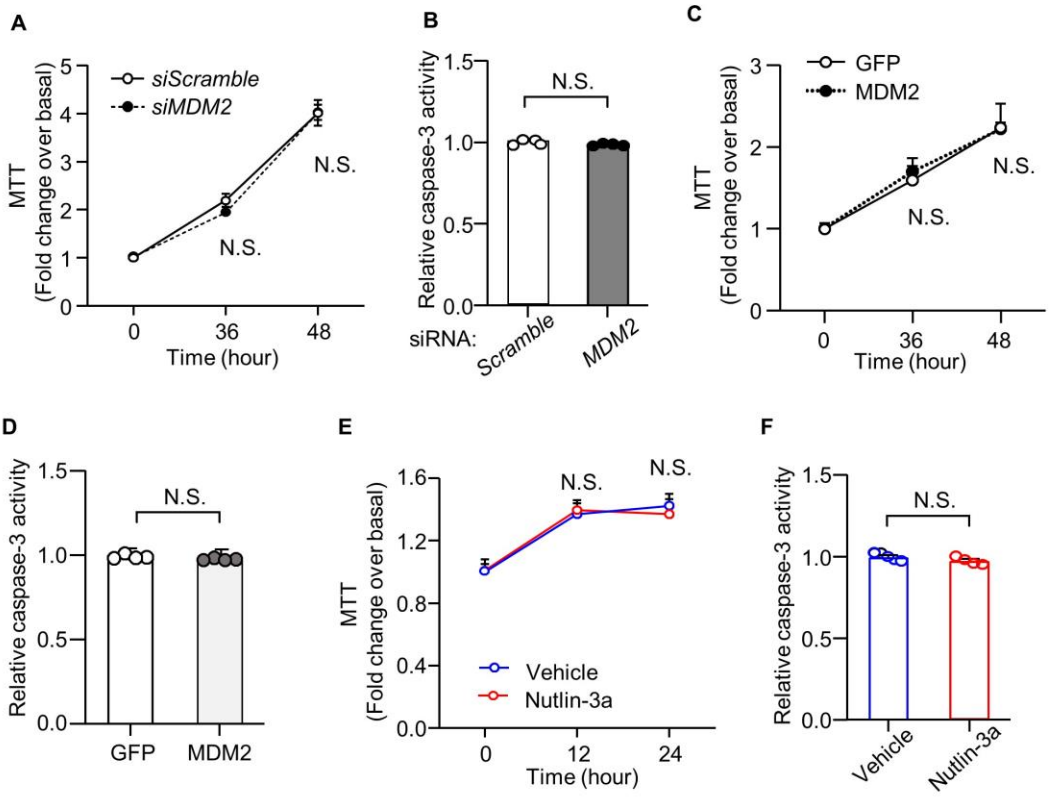

Figure S7. Effect of pharmacological or genetic modulation of MDM2 on cell proliferation and apoptosis in HepG2 cells. (A-B) HepG2 cells were transfected with siRNA against *MDM2* or *Scramble* for 48 hours (n=4). (C-D) HepG2 cells were transfected with plasmid overexpressing GFP or MDM2 for 48 hours (n=4). (E-F) HepG2 cells were treated with 10 μM Nutlin-3a or DMSO (as vehicle control) for 24 hours (n=4). MTT assays in panel A, C and E. Caspase 3 activity in cell lysates normalized with protein concentration and expressed as fold change over their corresponding controls are shown in panel B, D and F. All data are presented as mean ± SEM. (Two-tailed independent Student's t test). Not statistical significance (N.S.).

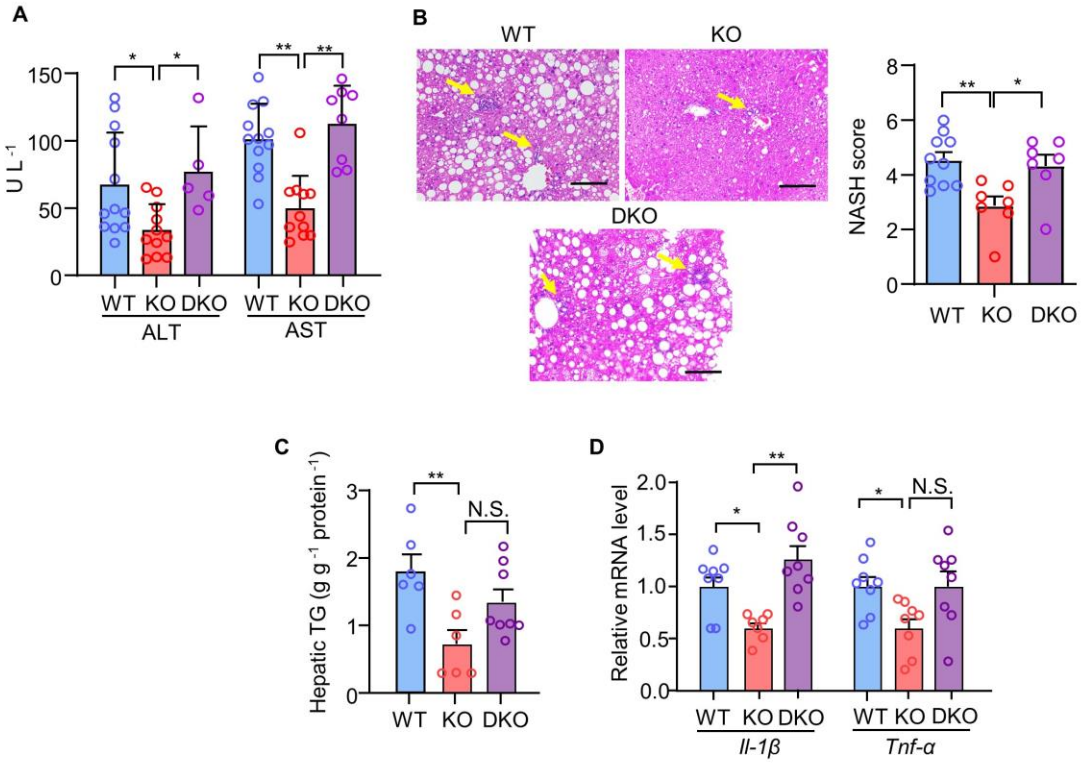

Figure S8. The severity of MAFLD is similar between DKO and their WT littermates but is alleviated in KO mice under HFHC feeding condition. DKO, KO and their WT littermates fed HFHC diet for 16 weeks were used. (A) Circulating levels of ALT and AST (n=5-12). (B) H&E staining of liver sections. Scale bar: 100 µm. Yellow arrows indicate immune cell clusters. The right panel is NASH score (n=7-10). (C) Hepatic TG levels determined by biochemical method (n=6-8). (D) mRNA expressions of hepatic *Il-1β* and *Tnf-α* normalized with average of *18s* and *36B4* (n=7-8). Representative images are shown. All data are presented as mean ± SEM. *p< 0.05 and **p<0.01. (One-way ANOVA with Tukey test). Not statistical significance (N.S.)

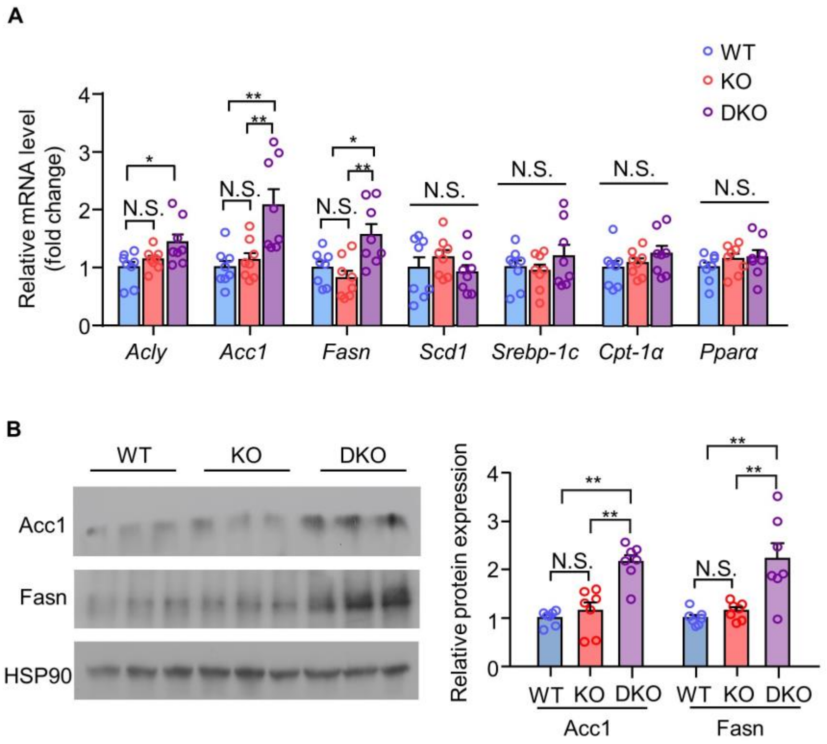

Figure S9. The lipogenic enzymes are upregulated in liver of DKO mice. Livers collected from DKO, KO and their WT littermates fed HFHC diet for 16 weeks were analyzed. (A) mRNA levels of *Fasn*, *Acc1*, *Acly*, *Scd1*, *Srebp-1c*, *Cpt-1α* and *Pparα* were detected and normalized with *18s* and *36B4* (n=8). (B) Immunoblotting analysis of FASN, ACC1 and HSP90. The bar chart is densitometric analysis of FASN and ACC1 normalized with HSP90 (n=7). All data are presented as mean ±SEM. *p< 0.05 and **p<0.01. (One-way ANOVA with Tukey test). Not statistical significance (N.S)

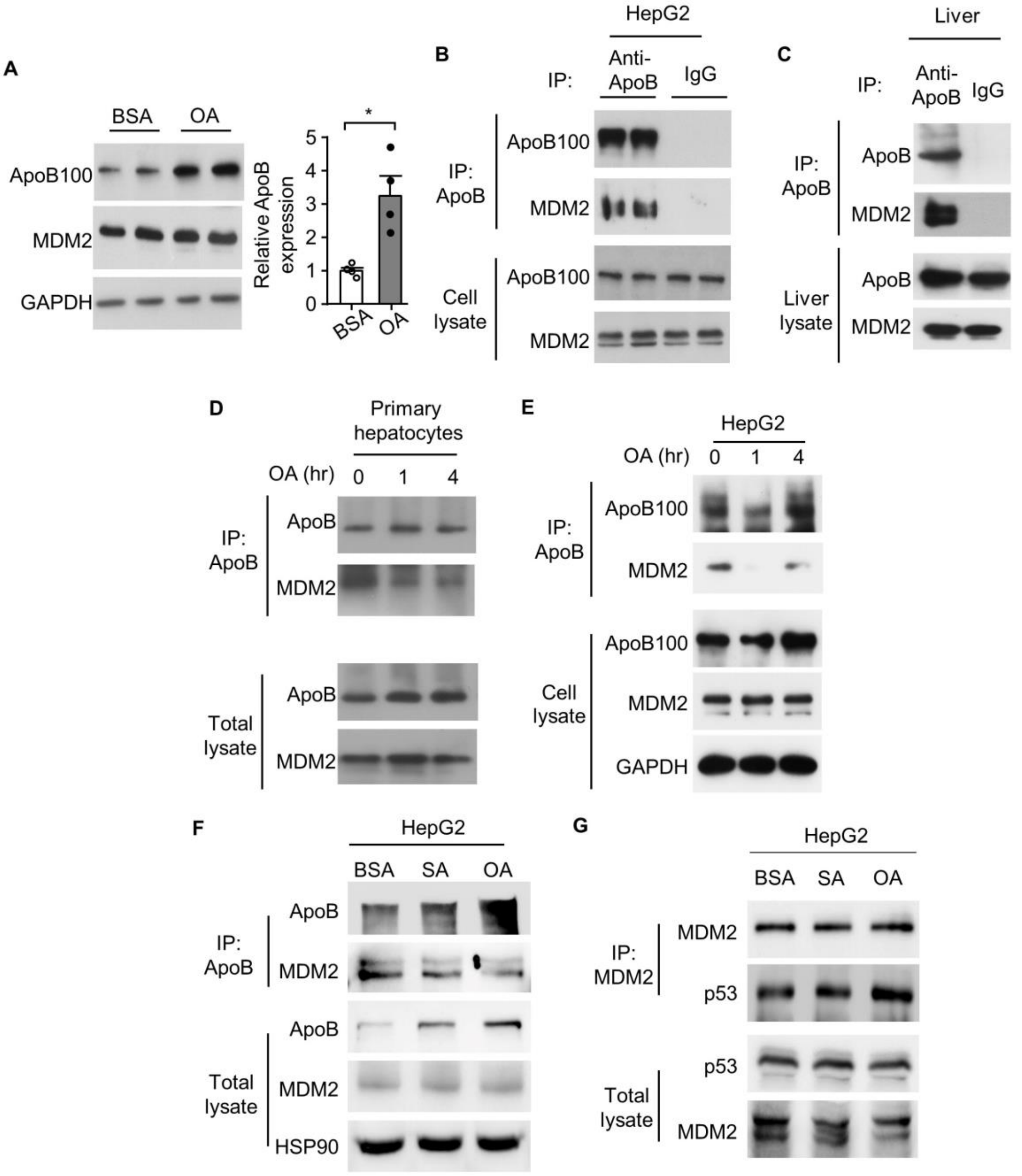

Figure S10. MDM2 interacts with ApoB in hepatocytes, and the interaction is suppressed by non-saturated free fatty acids. (A) HepG2 cells were treated with 0.4 mM OA or BSA as control for 8 hours, followed by immunoblotting analysis as indicated. The bar chart is densitometric analysis of ApoB normalized with GAPDH (n=4). (B) Total cell lysates from HepG2 cells and (C) liver of C57BL/6J mice were subjected to immunoprecipitation (IP) of ApoB, following by immunoblotting analysis of ApoB and MDM2. (D) Primary hepatocytes and (E) HepG2 cells were treated with 0.4 mM OA or BSA for 1 or 4 hours as indicated. (F-G) HepG2 cells were treated with 0.4 mM OA, 0.4 mM stearic acid (SA) or BSA for 4 hours. (D-F) Total lysates were subjected to immunoprecipitation of ApoB, followed by immunoblotting analysis of ApoB and MDM2. (G) Total lysates were subjected to immunoprecipitation (IP) of MDM2, followed by immunoblotting analysis of MDM2 and p53. Representative immunoblotting images are shown from three independent experiments. Data are expressed as mean ± SEM. *$p < 0.05$ (Independent Student's t test)

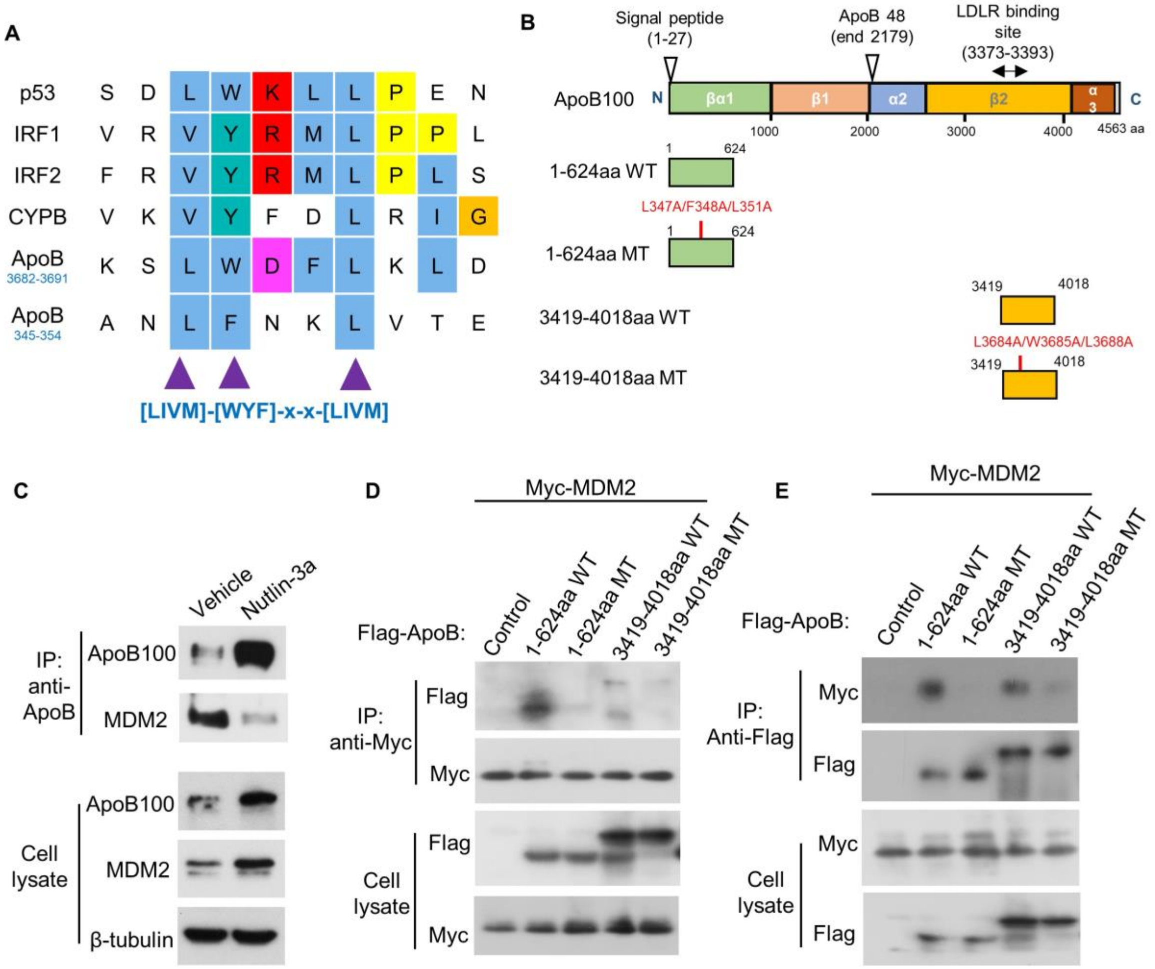

Figure S11. The interaction of MDM2 and ApoB is mediated by MDM2 interaction binding consensus motifs. (A) In silico binding site analysis. The consensus motifs ([LIVM]-[WYF]-x-x-[LIVM]) are responsible for bindings of MDM2 with p53, interferon regulatory factor 1 (IRF1), IRF2, cyclophilin B (CYPB), and ApoB proteins. L: leucine, I: isoleucine; V: valine; M: methionine; W: tryptophan; Y: tyrosine; F: phenylalanine; X: any amino acid. This motif ([LIVM]-[WYF]-x-x-[LIVM]) in a MDM2 interaction consensus was found by Judith Nicholson et al. based on Nutlin-3a responsiveness in a cell-based proteomics screening. Potential MDM2 binding sites in ApoB was predicted using a ClustalW sequence alignments program. Potential MDM2 binding motifs in ApoB were presented using Clustal colouring method. (B) Construction of Flag-tagged truncated ApoB expression plasmids containing the MDM2 binding motifs and their mutants (MT). The L, W and L in the MDM2 binding motif in ApoB were mutated to alanine (A) as indicated. (C) HepG2 cells after treatment with Nutlin-3a or vehicle were subjected to immunoprecipitation (IP) using an antibody against ApoB, followed by immunoblotting as indicated. (D-E) HEK 293 cells were transfected with plasmids encoding Myc-tagged MDM2 and FLAG-tagged truncated ApoB and their mutants for 48 hours. Total cell lysates were subjected to IP using (D) anti-Myc or (E) anti-FLAG antibodies, followed by immunoblotting analysis as indicated. Representative images are shown.

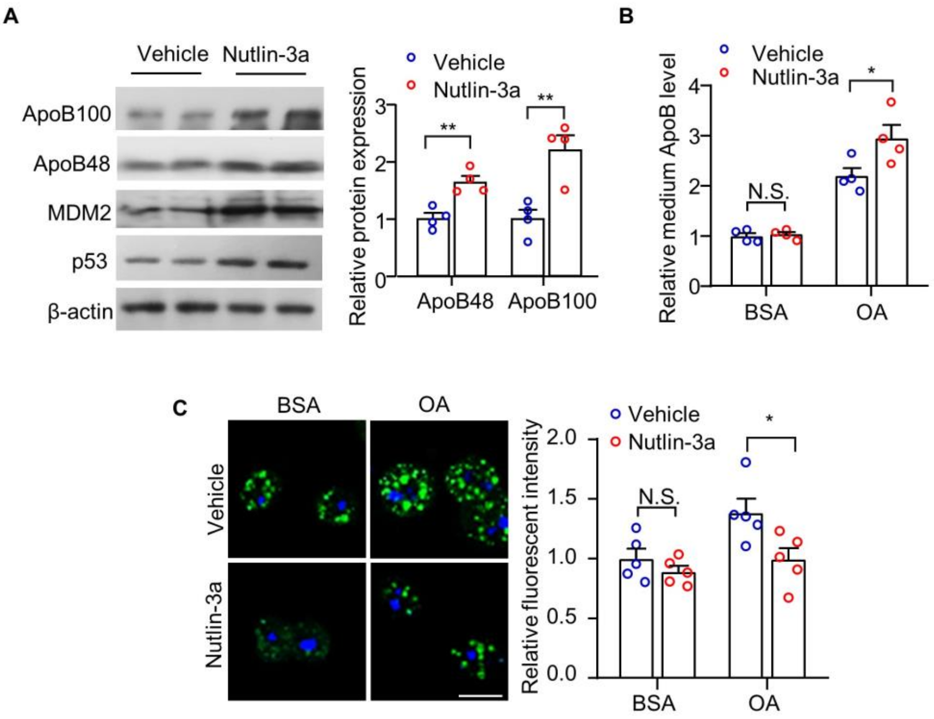

Figure S12. The MDM2 inhibitor Nutlin-3a enhances ApoB expression and reduces lipid accumulation in primary hepatocytes. Primary hepatocytes were isolated from 12-week-old male mice and treated with 20 μM Nutlin-3a or DMSO (as vehicle) for 24 hours, followed by treatment with 0.4 mM oleic acid (OA) conjugated with BSA or BSA as control for 8 hours. (A) The treated primary hepatocytes were subjected to immunoblotting analysis as indicated. The bar chart is the relative expressions of ApoB100 and ApoB48 normalized with β-actin (n=4). (B) Relative ApoB levels in culture medium normalized with protein concentration (n=4). (C) The cells were fixed and stained with BODIPY lipid probes (green) and DAPI (blue) (scale bar: 10 μm). The bar chart is the relative intensities of green fluorescence over cell size and each data point represents the average BODIPY intensity of hepatocytes in an area of interest (n=5). Representative images are shown. All data are presented as mean ± SEM. *$p < 0.05$; **$p < 0.01$. (Two-tailed independent Student's t test). Not statistical significance (N.S)

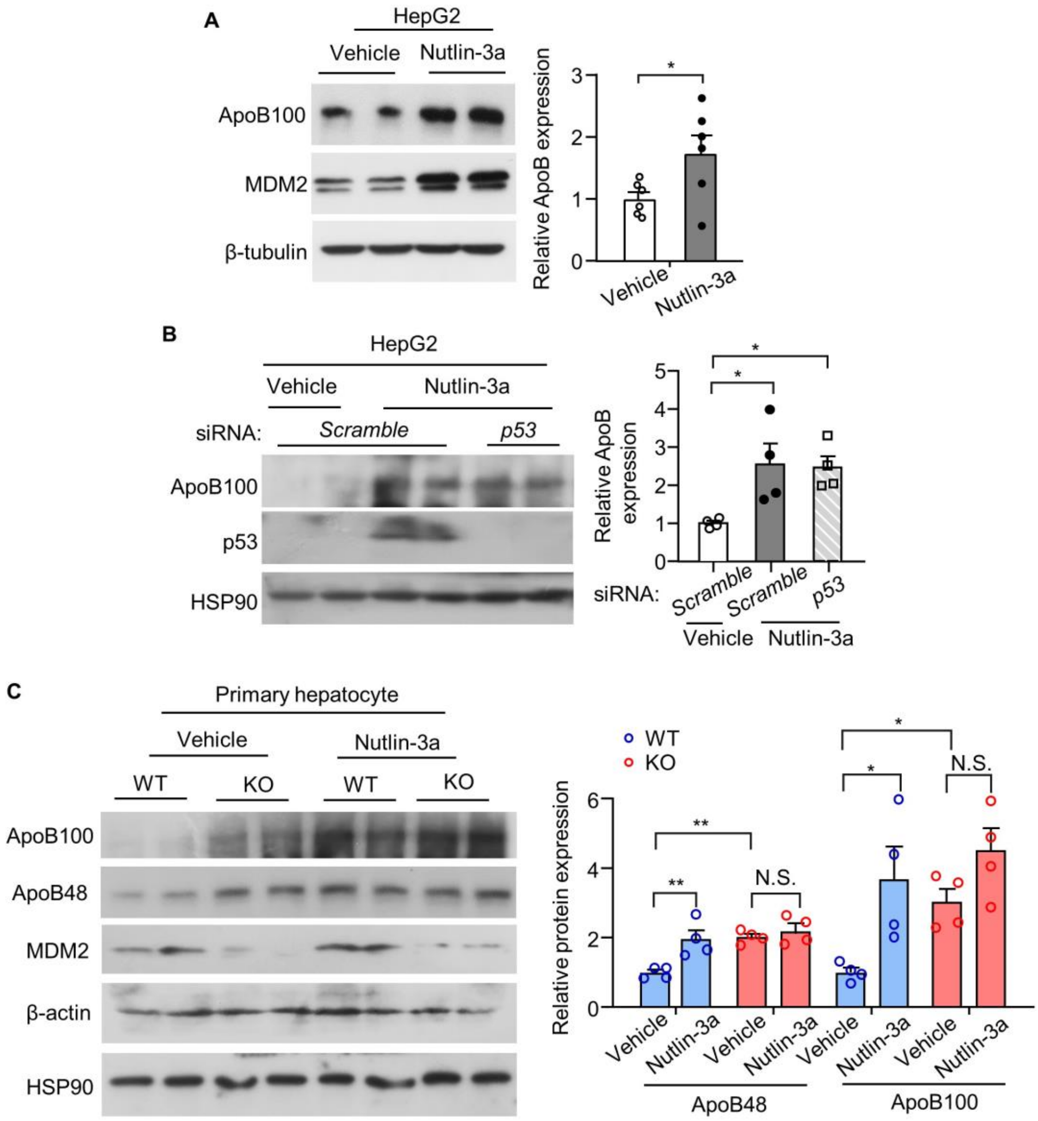

Figure S13. The promoting effect of Nutlin-3a on ApoB expression requires MDM2 but not p53 in hepatocytes. (A) HepG2 cells were treated with 10 μM Nutlin-3a or DMSO (as vehicle control) for 24 hours, followed by immunoblotting analysis as indicated. The bar chart is densitometric analysis for the relative ApoB100 protein abundances normalized with β-tubulin (n=6). (B) HepG2 cells were transfected with siRNA against *p53* or *Scramble* for 48 hours, followed by treatment with 10 μM Nutlin-3a or DMSO for 24 hours. The treated cells were subjected to immunoblotting analysis of ApoB100, p53 and HSP90. The right panel is densitometric analysis of ApoB normalized with HSP90 (n=4). (C) Primary hepatocytes isolated from 12-week-old male H-MDM2-KO and their WT littermates were treated with 20 μM Nutlin-3a or DMSO for 24 hours, followed by immunoblotting analysis as indicated. The bar chart is the relative expressions of ApoB100 and ApoB48 normalized with HSP90. (n=4). Representative immunoblot images are shown. All data are presented as mean ± SEM. *$p< 0.05$ and **$p<0.01$. (Two-tailed independent Student's test in panel A; One-way ANOVA with Tukey test in panel B or Two-way ANOVA in panel C). Not statistical significance (N.S).

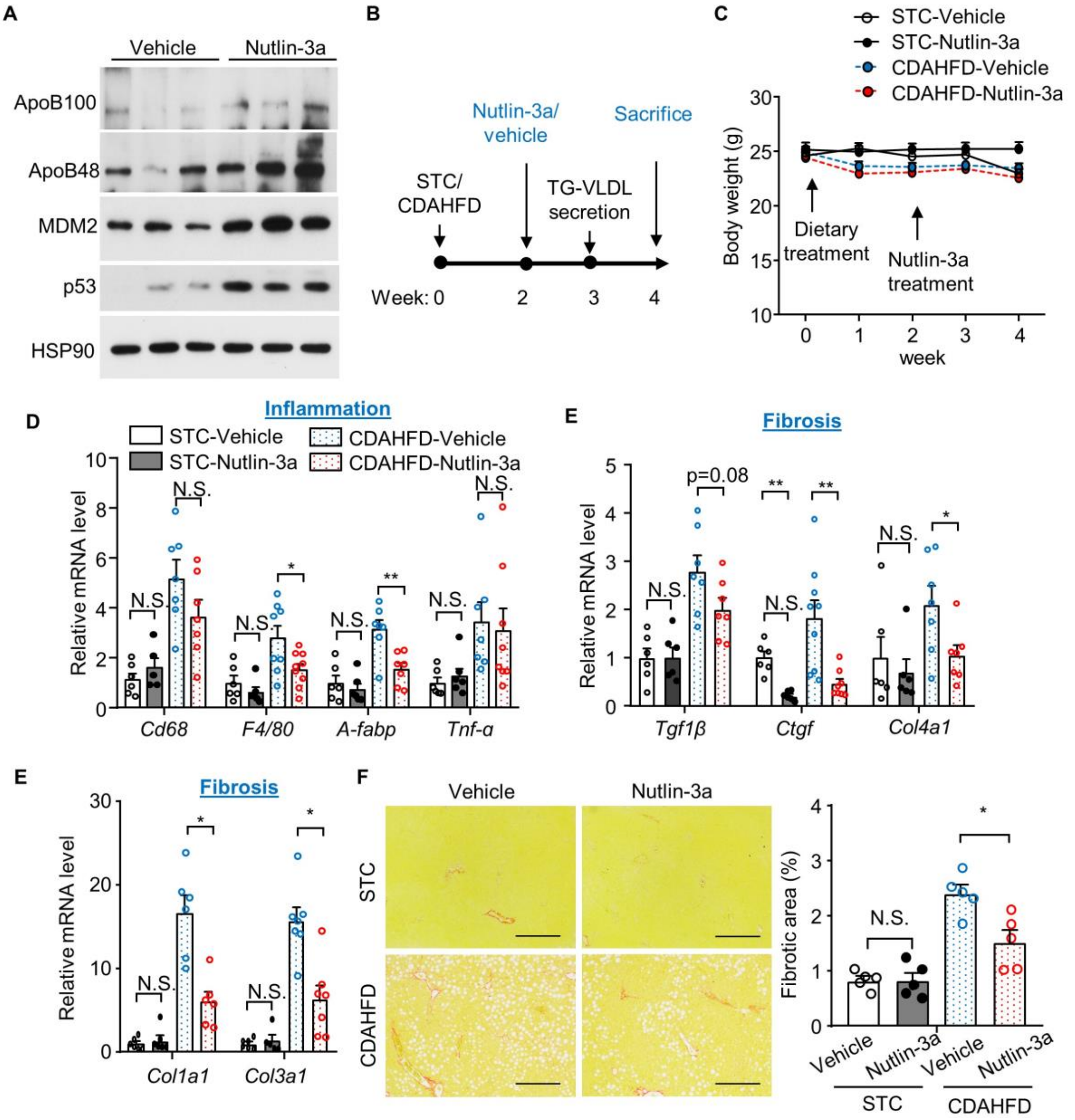

Figure S14. Treatment with Nutlin-3a alleviates CDAHFD-induced hepatic fibrosis and inflammation, but has no obvious effect on body weight. (A) 8-week-old C57BL/6J mice were injected with Nutlin-3a or vehicle twice a day for 3 days. The livers were collected for immunoblotting analysis of ApoB, MDM2, p53 and HSP90 in the liver. (B-D) 8-week-old C57BL/6J mice were fed with STC or CDAHFD for 4 weeks. (B) The first week of experiment is define as week-0. At week-2, the mice were treated with nultin-3a twice a day for two weeks. TG-VLDL secretion assay was performed at week-3 and the mice were sacrificed at week-4. (C) Body weight changes during the experimental period. (D-F) The liver samples were collected from the mice after treatment with Nutlin-3a for 2 weeks. (D-E) mRNA levels of *Cd68*, *F4/80*, *A-fabp*, *Tnf-a*, *Ctgf*, *Tgf-β*, *Col3α1*, *Col4α1* and *Col1α1* in the liver samples. (F) Picro Sirius Red staining in the liver sections. Scale bar: 200 µm. The bar chart is quantification of Picro Sirius Red positive area by ImageJ. All data are represented as mean ± SEM. n=5-6 for the STC group and n=5-9 for the CDAHFD group. *p< 0.05 and **p<0.01. (U test for *Tnf-a*-STC in panel D, and for *Col4a1*-STC, *Col1a1*-STC and *Col3a1*-STC in panel E; Welch's t-test for *Ctgf* in panel E; two-tailed independent Student's t test for the remaining data). Not statistical significance (N.S.).

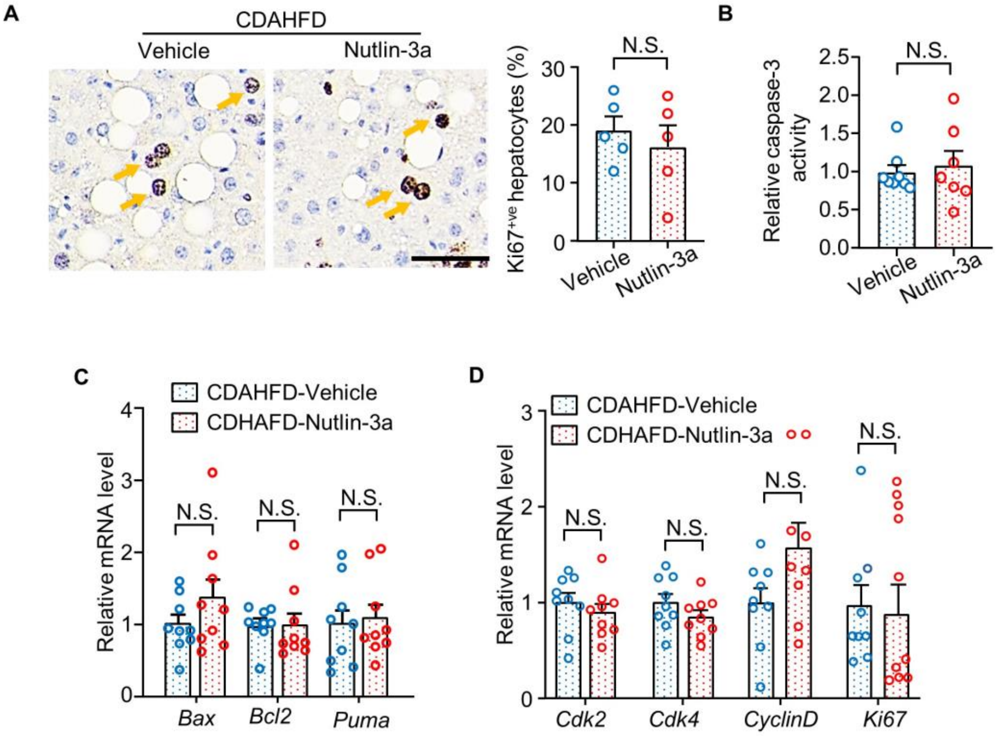

Figure S15. Treatment with Nutlin-3a has no obvious effect on cell proliferation and apoptosis in the liver. C57BL/6J mice were fed with CDAHFD diet for 2 weeks, followed by treatment with Nutlin-3a or DMSO (as vehicle control) for 2 weeks. Livers were collected for the following analyses. (A) Ki67 staining in liver sections. Yellow arrows indicate Ki67^{+ve} hepatocytes. Scale bar: 50 μm (n=5). (B) Activity of caspase-3 in liver lysates normalized with protein concentration and expressed as fold-change over vehicle control (n=7-8). (C-D) QPCR analysis of genes related to (C) cell apoptosis (*Bax, Bcl2* and *Puma*) and (D) cell proliferation (*Cdk2, Cdk4, CyclinD* and *Ki67*). The target genes were normalized with *18s* and expressed as fold-change over vehicle controls (n=9-10). All data are presented as mean ±SEM. (U test for *Bax* and *Bcl2* in panel C; Welch's t-test for *Ki67* in panel D; two-tailed independent Student's t test for the remaining data). Not statistical significance (N.S).

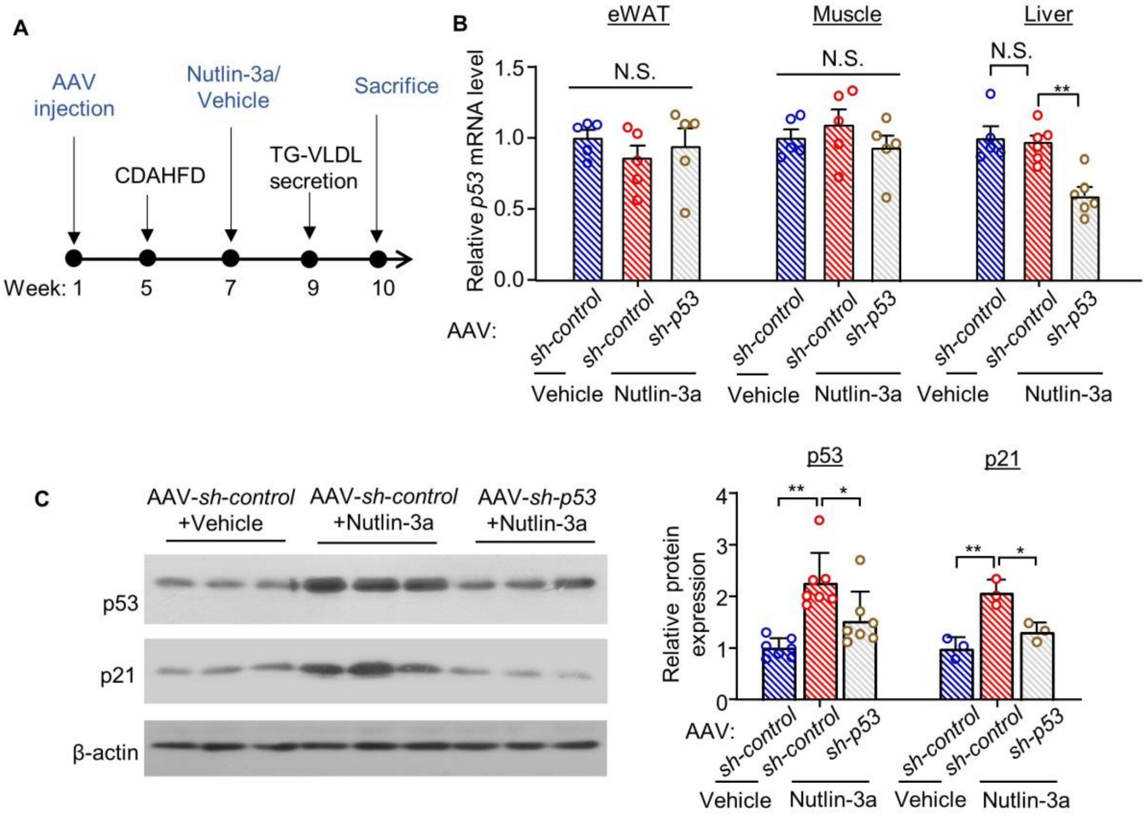

Figure S16. AAV-mediated knockdown of p53 in liver but not in adipose tissue and muscle. (A) 12-week-old male C57BL/6J mice were injected with AAV encoding shRNA against *p53* or *negative control*. The first week of AAV injection is defined as week-1. After 4 weeks of AAV injection, the mice were fed CDAHFD for two weeks, followed by treatment with Nutlin-3a or vehicle for 3 weeks. The mice were sacrificed at week-10. (B) mRNA expression of *p53* in eWAT, muscle and liver normalized with *β-actin* (n=5-6). (C) Representative immunoblots of p53, p21 and β-actin in livers. The right panel is densitometric analysis of p53 and p21 normalized with β-actin (n=3 for p21 and 7 for p53). All data are presented as mean ± SEM. $*p < 0.05$, $**p < 0.01$. (One-way ANOVA with Tukey test). Not statistical significance (N.S.).

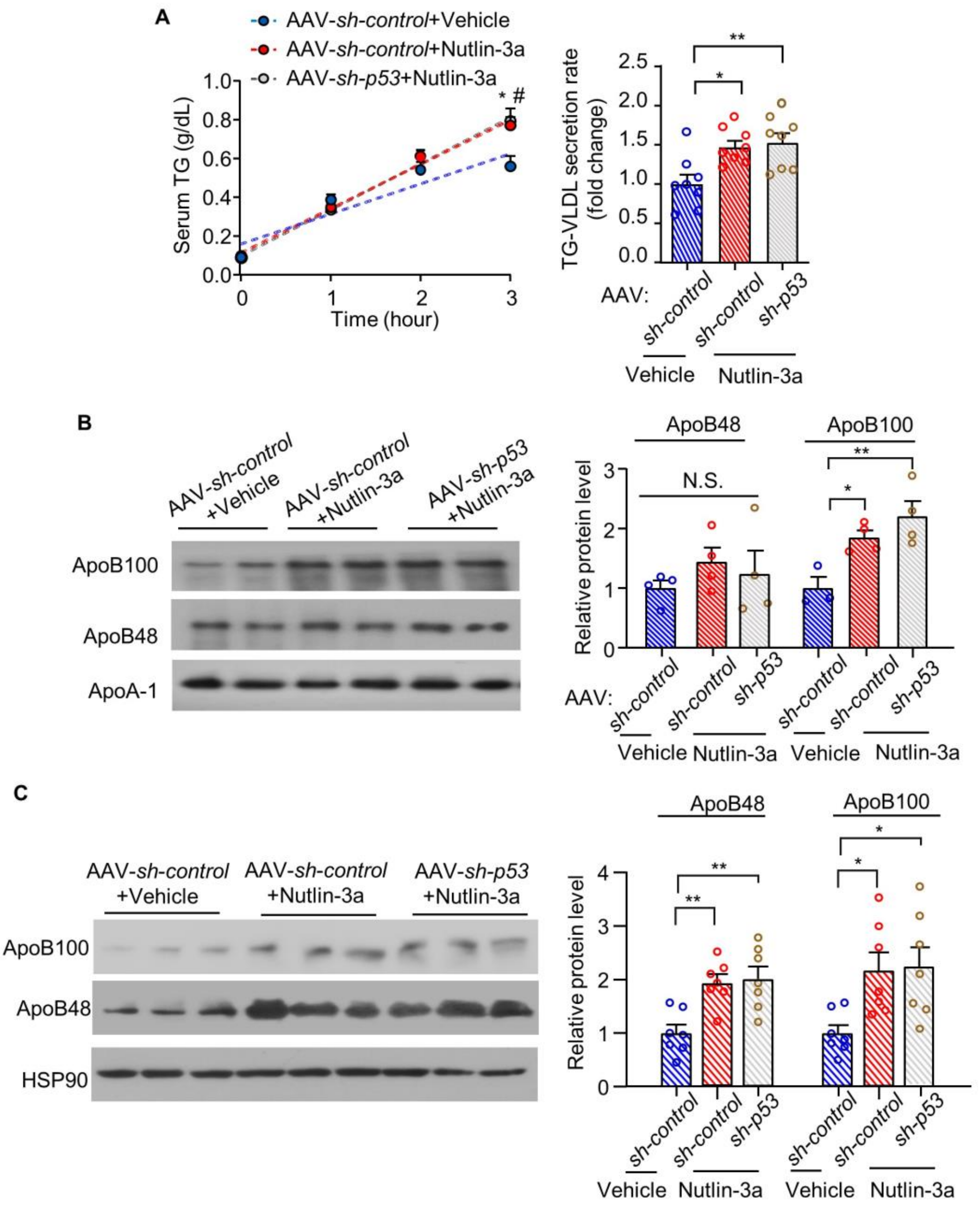

Figure S17. The upregulating effects of Nutlin-3a on ApoB expression and VLDL-TG secretion are independent of p53. Animal model and treatment scheme are described in Figure S16A. (A) *In vivo* TG-VLDL secretion assay was performed at week-9. The bar chart is the fold change of TG-VLDL secretion rate over mice treated with AAV-*sh-control* + Vehicle (n=8). (B) Immunoblotting analysis of serum ApoB and ApoA-1 after tyloxapol injection for 3 hours. The right panel is densitometric analysis of ApoB100 and ApoB48 normalized with ApoA-1 (n=3-4). (C) Immunoblotting analysis of ApoB in livers. The right panel is densitometric analysis of ApoB100 and ApoB48 normalized with HSP90 (n=7). All data are presented as mean ± SEM. *p< 0.05 and **p<0.01. (One-way ANOVA with Tukey test). Not statistical significance (N.S.).

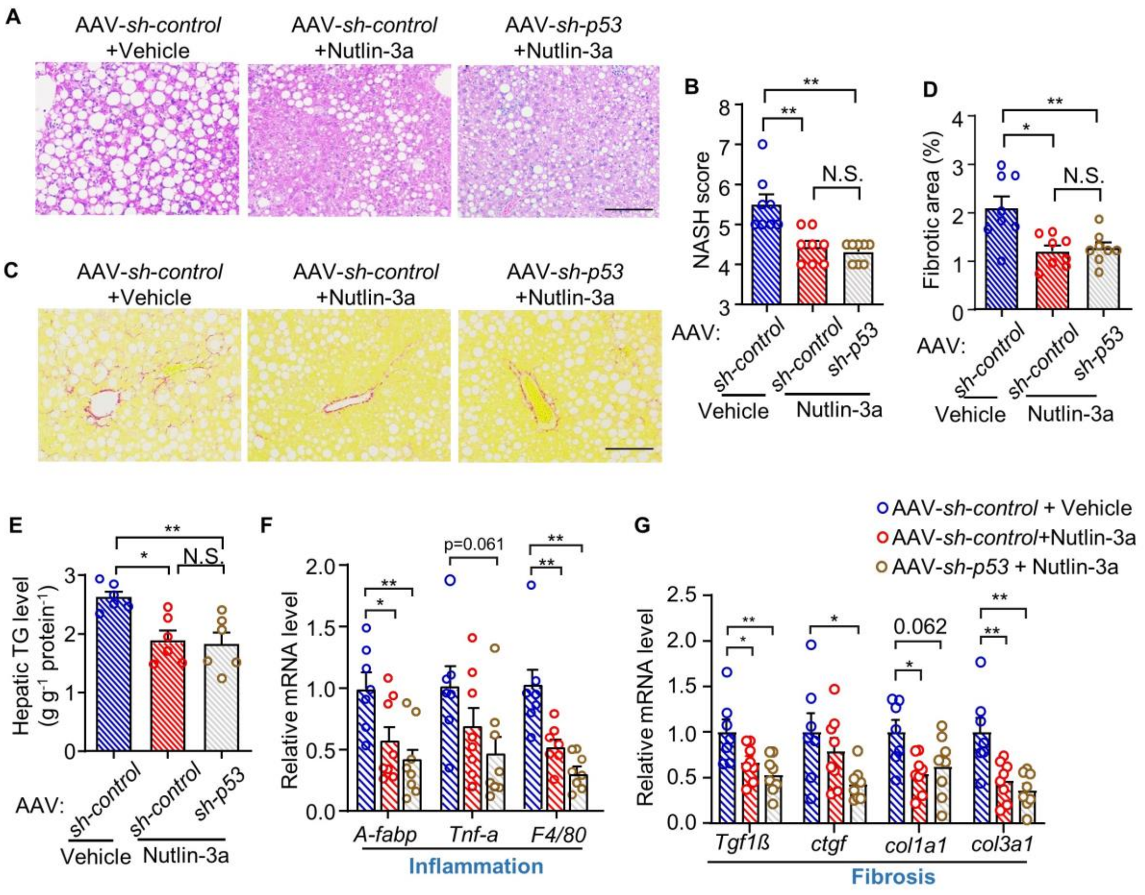

Figure S18. The hepatoprotective effects of Nutlin-3a are independent of p53. Animal model and treatment scheme are described in Figure S16A. The livers were collected at week-10 for the following analyses. (A) Representative images of H&E staining of liver sections. Scale bar: 100 µm. (B) NASH score in liver sections in panel A (n=8). (C) Representative images of Picro Sirius Red staining in liver sections. Scale bar: 100 µm. (D) The bar chart is quantification of Picro Sirius Red positive area by ImageJ (n=8). (E) Hepatic TG contents determined by biochemical assay (n=6). (F-G) QPCR analysis of genes related to (F) inflammation and (G) fibrosis in liver samples (n=7-8). All data are represented as the mean ± SEM. *p< 0.05 and **p<0.01. (Kruskal Wallis test for panel B and *F4/80* in panel F; One-way ANOVA with Tukey test for the remaining data). Not statistical significance (N.S.).

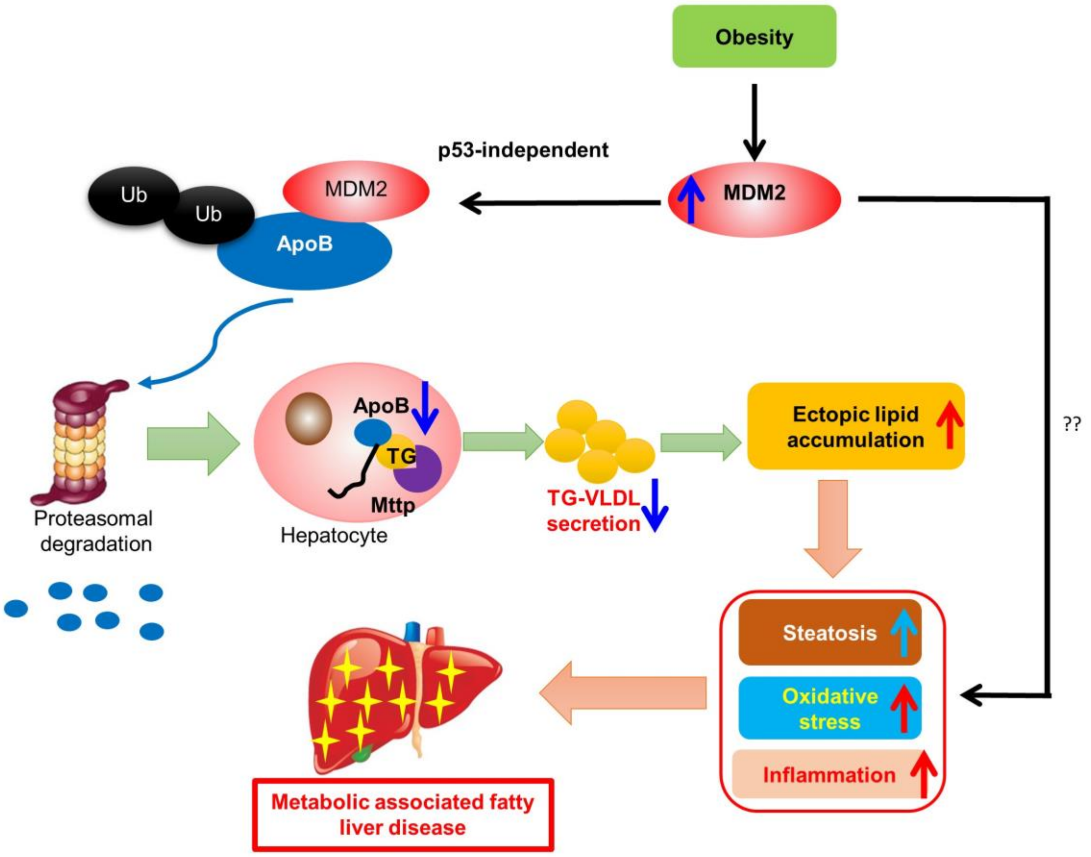

Figure S19. Working model: Overexpression of MDM2 triggers MAFLD by blocking TG-VLDL secretion in hepatocytes. Obesity increases hepatic MDM2 protein expression, which interacts with ApoB and promotes its proteasomal degradation via the lysine 48-linked polyubiquitination. Reduction of ApoB protein leads to defective TG-VLDL secretion, resulting in lipotoxicity and MAFLD. The regulatory action of MDM2 on ApoB expression is independent of p53 pathway.

	Normal liver (n=16)	Steatosis (n=29)	NASH (n=28)	P-value Normal liver Vs Steatosis	P-value Normal liver Vs NASH	P-value Steatosis Vs NASH
Age, year	29.58±2.9	30.37±1.67	28.81±1.97	>0.999	>0.999	>0.999
Men, %	18.75	41.38	57.14	0.123	0.013	0.234
BMI [kg m^{-2}]	36.5±1.62	40.69±1.92	44.53±1.69	0.412	0.006	0.175
Waist [cm]	113.38±3.51	123.41±3.71	130.23±3.45	0.187	0.010	0.615
ALT [U L^{-1}]	21.03±2.82	64.64±12.73	79.86±10.86	0.005	<0.0001	0.158
AST [U L^{-1}]	17.57±1.12	40±6.8	50.93±7.49	0.009	<0.0001	0.114
TCHOL [mg dL^{-1}]	1.27±0.09	1.83±0.35	2.12±0.19	>0.999	0.254	0.421
TG [mg dL^{-1}]	4.77±0.26	5.01±0.17	5.28±0.16	0.382	0.002	0.086
HDL-C [mg dL^{-1}]	1.13±0.06	1.05±0.03	0.96±0.04	0.202	0.030	0.903
LDL-C [mg dL^{-1}]	2.93±0.19	3.07±0.12	3.38±0.12	>0.999	0.169	0.155
APOA [mg dL^{-1}]	1.29±0.05	1.26±0.04	1.2±0.04	0.540	0.528	>0.999
APOB [mg dL^{-1}]	0.94±0.06	1.01±0.04	1.16±0.04	>0.999	0.026	0.064

Table S1. Characteristics of the study participants. ALT, alanine aminotransferase; AST, aspartate transaminase; TCHOL, total cholesterol; TG, triglyceride; HDL-C, high-density lipoprotein cholesterol; LDL-C, low-density lipoprotein cholesterol; ApoA, apolipoprotein A; ApoB, apolipoprotein B. All data are presented as the mean ± SEM. Kruskal Wallis test for the comparison among the three groups except gender.

	WT-STC	KO-STC	WT-HFHC	KO-HFHC
Triglyceride [mg dL^{-1}]	61.23±7.06	58.95±3.69	124.01±11.45	116.43±5.72
Cholesterol [mg dL^{-1}]	41.98±2.86	47.87±3.04	135.14±18.77	112.53±4.55
Glucose [mM]	4.83±0.29	4.64±0.24	6.63±0.49	6.82±0.40
FFA [mM]	0.64±0.04	0.68±0.01	1.19±0.32	1.22±0.31
β-hydroxybutyrate [mM]	1.03±0.17	1.05±0.29	0.97±0.29	0.35±0.27*
Liver weight [g]	1.19±0.04	1.25±0.03	2.60±0.35	2.13±0.31

Table S2. Serum profile and liver weight in H-MDM2-KO mice and their WT littermates. H-MDM2-KO mice and WT littermates were fed with a STC or a HFHC diet for 16 weeks and serum samples were collected after fasting for 24 hours. Serum levels of triglyceride, cholesterol, free fatty acids (FFA) and glucose were determined using enzymatic kits or glucose meter (n=8). The concentration of circulating adiponectin was measured using an adiponectin ELISA kit (n=3-4 for STC group and n=6 for HFHC group). The wet liver weight was measured (n=5-7 for the STC group, n=12 for the HFHC group). *$p<0.05$; KO-HFHC vs WT-HFHC. All data are represented as the mean ± SEM. (U test for cholesterol-HFHC and FFA-STC; two-tailed independent Student's t test for the remaining data).

	WT-HFHC	DKO-HFHC
Body weight [g]	38.9 ± 2.9	41.0 ± 3.8
Liver weight [g]	1.86 ± 0.42	1.70 ± 0.49
Fat mass [g]	10.82 ± 1.89	13.19 ± 1.57

Table S3. Body weight, liver weight and fat mass of DKO mice and their WT littermates under HFHC feeding condition. DKO mice and their WT littermates fed with HFHC diet for 16 weeks were used. All data are represented as the mean ± SEM. *$p < 0.05$. (Two-tailed independent Student's t test).

	STC-Vehicle	STC-Nutlin-3a	CDAHFD-Vehicle	CDAHFD-Nutlin-3a
Triglyceride [mg dL^{-1}]	64.53±6.86	71.56±3.76	59.53±3.83	78.08±4.22$^{\#}$
Cholesterol [mg dL^{-1}]	193.07±11.18	208.23±4.26	113.71±4.01	137.95±3.14$^{\#}$
Glucose [mM]	8.88±0.45	10.15±0.36	7.22±0.30	7.36±0.26
ALT [U L^{-1}]	27.89±2.75	19.96±1.06*	159.33±14.18	86.13±11.58$^{\#}$
AST [U L^{-1}]	41.85±5.41	33.39±5.80	158.26±11.89	84.89±10.12$^{\#}$
β-hydroxybutyrate [mmol L^{-1}]	1.34±0.13	1.13±0.25	1.97±0.18	1.58±0.21
Liver weight [g]	1.09±0.04	1.15±0.01	1.44±0.04	1.44±0.04
Food intake [g $mouse^{-1}$ day^{-1}]	3.329±0.02	3.836±0.07	3.40±0.10	3.39±0.60

Table S4. Serum profile and liver weight in mice treated with Nutlin-3a. Serum were collected in C57BL/6J mice after 1 week treatment of Nutlin-3a or vehicle as described in Figure S14B. Circulating levels of triglyceride, cholesterol, glucose, AST and ALT were measured under fed condition. Liver weight was measured after 2 weeks treatment of Nutlin-3a. Food intake per day during the whole experiment period was monitored and averaged. n=6 for the STC group and n=9 for the CDAHFD group. All data are represented as the mean ± SEM. (One-way ANOVA), *p< 0.05, STC-vehicle vs STC-Nutlin-3a group; #p< 0.05, CDAHFD-vehicle vs CDAHFD-Nutlin-3a. (U test for cholesterol-STC and food intake-HFHC; two-tailed independent Student's t test for remaining data).

	CDAHFD		
	***siNegative* +vehicle**	***siNegative* +Nutlin-3a**	***siApoB* +Nutlin-3a**
Triglyceride [mg dL^{-1}]	49.25±3.56	85.95±5.48*	22.07±1.95$^{\#}$
Cholesterol [mg dL^{-1}]	71.94±2.63	83.13±1.37*	42.37±2.11$^{\#}$
Glucose [mM]	8.33±0.25	8.12±0.22	8.52±0.33
ALT [U L^{-1}]	220.00±6.02	165.75±17.07*	235.76±13.80$^{\#}$
AST [U L^{-1}]	153.26±7.28	118.19±10.40*	174.86±8.66$^{\#}$
Liver weight [g]	1.61±0.05	1.64±0.04	1.80±0.05
Food intake [g $mouse^{-1}$ day^{-1}]	3.19±0.19	3.32±0.17	3.18±0.01

Table S5. Serum profile and liver weight in mice treated with siRNA against *ApoB*. 8-week-old male C57BL/6J mice fed with CDAHFD were treated with Nutlin-3a or vehicle and/or injection with siRNA against *ApoB (siApoB)* or negative control (*siNegative*) as described in Figure 8A. Serum samples were collected at day 20 before VLDL secretion assays for measurement of triglyceride, cholesterol, glucose, ALT and AST. Wet liver weight was measured after the mice were sacrificed (n=6). All data are represented as the mean ± SEM. *$p< 0.05$, *siNegative*+vehicle vs *siNegative*+Nutlin-3a; #$p<0.05$, *siNegative*+Nutlin-3a vs *siApoB*+Nutlin-3a. (One-way ANOVA with Tukey test),

	CDAHFD		
	AAV-*sh-control*+ Vehicle	AAV-*sh-control*+ Nutlin-3a	AAV-*sh-p53*+ Nutlin-3a
Triglyceride [mg dL^{-1}]	45.14±5.30	68.56±6.47*	68.76±7.86#
Cholesterol [mg dL^{-1}]	102.06±3.74	105.38±3.31	101.97±6.01
Glucose [mM]	8.64±0.52	9.48±0.45	9.35±0.40
ALT [U L^{-1}, week 8]	194.80±12.34	147.17±7.85**	155.06±7.29#
ALT [U L^{-1}, week 9]	172.83±12.83	111.91±14.51**	116.39±12.56##
AST [U L^{-1}]	206.67±38.80	170.46±34.87	114.08±22.83
Body weight [g]	25.52±0.40	25.00±0.40	25..77±0.65
Liver weight [g]	1.55±0.05	1.50±0.12	1.48±0.06
Food intake [g $mouse^{-1}$ day^{-1}]	2.51±0.10	2.56±0.12	2.56±0.16

Table S6. Serum profile and liver weight in mice injected with AAV encoding shRNA against *p53* or *negative control*. The animal model and treatment scheme are described in Figure S16A. Serum samples were collected at week-9 for measurement of triglyceride, cholesterol, glucose, ALT and AST. Wet liver weight at week 10 (n=8). All data are represented as mean ± SEM. *p< 0.05 and **p<0.01, AAV-*sh-control*+Vehicle vs AAV-*sh-control*+Nutlin-3a; #p<0.05 and ##p<0.01, AAV-*sh-control*+vehicle vs AAV-*sh-p53*+Nutlin-3a. (One-way ANOVA with Tukey test).

Gene	Sequence 5’-3’
siRNA against human *MDM2*	GCCACAAAUCUGAUAGUAU
siRNA against human *p53*	UGUUCCGAGAGCUGAAUGA
Scramble siRNA control in the cell study	UUCUCCGAACGUGUCACGUTT
sh-p53 in AAV	GGACAGCCAAGTCTGTTATGT
sh-control in AAV	AGGCAGAAGTATGCAAAGCAT

Table S7. Sequences of siRNA and shRNA used in this study.

Gene name	Forward primer (5'-3')	Reverse primer (5'-3')
Apob (m)	ACTGTGACTTCAATGTGGAGTATAA	CAGATGAAAGTCAGTCAGTGCT
Mttp (m)	AGTGCAGTTCTCACAGTACC	TTTCTTCTCCGAGAGACATATCC
Tnf-α (m)	CCCTCACACTCAGATCATCTTCT	GCTACGACGTGGGCTACAG
Mcp-1 (m)	CCACTCACCTGCTGCTACTCA	TGGTGATCCTCTTGTAGCTCTCC
A-fabp (m)	CCGCAGACGACAGGA	CTCATGCCCTTTCATAAACT
F4/80 (m)	CTTTGGCTATGGGCTTCCAGTC	GCAAGGAGGACAGAGTTTATCGTG
Cd68 (m)	TTGGGAACTACACACGTGGGC	CGGATTTGAATTTGGGCTTG
Il-1β (m)	CTGGTGTGTGACGTTCCCATTA	CCGACAGCACGAGGCTTT
Ctgf (m)	GTGCCAGAAAGCACACTG	CCCCGGTTACACTCCAAA
Col3α1 (m)	CTGTAACATGGAAACTGGGGAAA	CCATAGCTGAACTGAAAACCACC
Col4α1 (m)	CTGGCACAAAAGGGACGAG	ACGTGGCCGAGAATTTCACC
Tgf-β (m)	ATCGACATGGAGCTGGTGAA	TGTTGTACAAAGCGAGCACC
Fasn (m)	GGAGGTGGTGATAGCCGGTAT	TGGGTAATCCATAGAGCCGAG
Srebp-1c (m)	GAAGCTGTCGGGGTAGCGTCT	CTCTCAGGAGAGTTGGCACCTG
Dgat2 (m)	AGTGGCAATGCTATCATCATCGT	TCTTCTGGACCCATCGGCCCCAGGA
Scd1 (m)	TGCTATCGGGGTGTTAATGA	TCTTGTGGCATGGTTAATCCTA
Cd36 (m)	ACATTTGCAGGTCTATCTACG	AATGGTTGTCTGGATTCTGG
Mcd (m)	GATCGCAATGGGTGCTTTTGATAGAA	AGCTGATTGGCAATGTCTCCAGCAAA
Ppar-α (m)	GGATGTCACACAATGCAATTCGC	TCACAGAACGGCTTCCTCAGGT
Pgc-1α (m)	CCCTGCCATTGTTAAGACC	TGCTGCTGTTCCTGTTTTC
Acat1 (m)	CCCCATTGATTTTCCACTTG	AGCACAACCACA
Hmgcs2 (m)	ATACCACCAACGCCTGTTATGG	CAATGTCACCACAGACCACCAG
Hmgcl (m)	ACTACCCAGTCCTGACTCCAA	TAGAGCAGTTCGCGTTCTTCC
Bhd1 (m)	GAAGATGCTGTCCGGCTAAG	CACCACTGGTCTGTTTGCAG
Pc (m)	GGGATGCCCACCAGTCACT	CATAGGGCGCAATCTTTTTGA
Pepck (m)	CTGCATAACGGTCTGGACTTC	CAGCAACTGCCCGTACTCC
G6pc (m)	ACTGTGGGCATCAATCTCCTC	CGGGACAGACAGACGTTCAGC

Table S8. Primer sequences for real-time quantitative PCR analysis in this study. The letter in the brackets refers to the gene origin. (m) refers to mouse and (h) refers to human.

Gene name	Forward primer (5’-3’)	Reverse primer (5’-3’)
18s (m)	AGTCCCTGCCCTTTGTACACA	CGATCCGAGGGCCTCACTA
Actin (m)	CTAAGGCCAACCGTGAAAAG	ACCAGAGGCATACAGGGACA
Col1a1(m)	GGTTGGGACAGTCCAGTTCT	CAATGGTGAGACGTGGAAAC
Cpt-1 α (m)	CTCCGCCTGAGCCATGAAG	CACCAGTGATGATGCCATTCT
ACLY (m)	ACCCTTTCACTGGGGATCACA	GACAGGGATCAGGATTTCCTTG
ACC1 (m)	ACAGTGGAGCTAGAATTGGAC	ACTTCCCGACCAAGGACTTTG
p53 (m)	ACTGCATGGACGATCTGTTG	GTGACAGGGTCCTGTGCTG
Ki67(m)	ATCATTGACCGCTCCTTTAGGT	GCTCGCCTTGATGGTTCCT
Puma(m)	ATGGCGGACGACCTCAAC	AGTCCCATGAAGAGATTGTACATGAC
Bak (m)	CAGCTTGCTCTCATCGGAGAT	GGTGAAGAGTTCGTAGGCATTC
Bax(m)	AGACAGGGGCCTTTTTGCTAC	AATTCGCCGGAGACACTCG
Cdk(m)	GCGACCTCCTCCCAATATCG	GTCTGATCTCTTTCCCCAACTCT
Cdk4(m)	CTGGAAGAAGTCTGCGTCGG	GTCTTGCCAAAGCGGTTCAG
Cyclin D (m)	GCAAGGATGGCAAGGATTGA	GCAATTCTGCCTGGATAGC
Ki67(h)	AGAAGAAGTGGTGCTTCGGAA	AGTTTGCGTGGCCTGTACTAA
Bax(h)	CCCGAGAGGTCTTTTTCCGAG	CCAGCCCATGATGGTTCTGA
PUMA(h)	GACCTCAACGCACAGTACGAG	AGGAGTCCCATGATGAGATTGT
CDK4 (h)	TCAGCACAGTTCGTGAGGTG	GTCCATCAGCCGGACAACAT
Cyclin D (h)	CAATGACCCCGCACGATTTC	CATGGAGGGCGGATTGGAA
ApoB (h)	ACCGGGGACACCAGATTAGA	AGGGTATCCACCAAGGCTCT
MTTP (h)	TCCAGGGTGGTCTAGCTAT	CCTTGTCCATCTGCATGCA
Actin (h)	GTCTTCCCCTCCATCGTG	GTACTTCAGGGTGAGGATGC

Table S8. Primer sequences for real-time quantitative PCR analysis in this study. The letter in the brackets refers to the gene origin. (m) refers to mouse and (h) refers to human.
